# The many manifestations of magical thinking: a systematic review

**DOI:** 10.3389/fpsyt.2026.1759906

**Published:** 2026-05-20

**Authors:** Clare M. Eddy

**Affiliations:** 1 College of Medicine and Health, University of Birmingham, Birmingham, United Kingdom; 2 Research and Development, Birmingham and Solihull Mental Health NHS Foundation Trust (BSMHFT), National Centre for Mental Health, Birmingham, United Kingdom

**Keywords:** agency, culture, magical thinking, obsessive compulsive disorder, schizophrenia, social cognition, spirituality, thought action fusion

## Abstract

Magical thinking (MT) involves beliefs that thoughts or actions can influence events in unrealistic ways. While MT is integral to obsessive compulsive disorder, and reflected in the cognitive features of schizophrenia, it is observable across the general population in various forms. Given its prevalence and potential relevance to a range of psychiatric conditions, understanding more about what may predispose an individual to MT, and how it may in some cases culminate in psychological distress or dysfunction would be helpful. This paper reports a systematic review of studies investigating MT, encompassing both magical ideation and thought-action fusion specifically, across the disciplines of psychiatry and psychology, to shed further light on the likely predisposing factors and behavioural consequences of MT, its potential neurobiological underpinnings, and role in psychiatric symptomatology. After exclusions, 191 studies were identified that explored MT in association with a diverse array of secondary topics, from gambling compulsions to childhood trauma, within both clinical and non-clinical samples, across a range of cultural contexts. On an intra-individual level, MT demonstrates numerous cognitive and emotional correlates, and on a societal level it may influence both social custom and religious tradition. A synthesis of the available evidence uncovers unexplored relationships with social cognition and mental health, and future research investigating its emerging relationships with stress, mood and social connection, may uncover functions beyond those exhibited by a simple marker of psychopathology.

## 1 Introduction

The term magical thinking (MT) is used to refer to judgments about causal relationships that do not follow rational laws or known scientific principles, an example of which may be a belief in “mind over matter”. One specific type of MT is Thought action fusion (TAF), which treats thoughts and actions as equivalent (1, 2). TAF includes the belief that thinking about a specific event makes it more likely to occur (TAF likelihood: TAF-L), and that thinking about doing something that is morally wrong is equivalent to performing that morally wrong action (TAF moral: TAF-M). Magical ideation (MI) is another construct that falls under the MT umbrella, as supported by the finding that scales designed to assess TAF and MI tend to correlate (e.g. (3, 4)). MI is reflected in certain kinds of beliefs, such as belief in the existence of telepathy, clairvoyance and spiritual influences, or in the predictive power of astrology or superstitions (5).

MT underpins a range of beliefs and behaviours spanning a continuum from normal to pathological. Within the clinical domain, links have been drawn between TAF and the symptoms of obsessive compulsive disorder (OCD). Indeed, the diagnostic criteria and assessments for OCD involve components of MT. Examples of how MT manifests in OCD include symptoms such as excessive worries about contamination being spread across objects, avoiding ‘bad’ numbers, and fear of harm coming to the self or others, resulting in rituals to reduce the likelihood of this (3, 6). While agency, responsibility, and the power of one’s own intentions appear central to OCD, there is also a strong element of morality, as well as associated distress. Beyond OCD, other psychiatric conditions that involve MT include schizophrenia spectrum disorders (SSDs), which feature MI in the form of beliefs about thought transference as well as auditory hallucinations (7, 8), and other symptoms suggestive of atypical attribution of agency, intention and causal relationships, such as ideas of reference.

Outside of the clinical domain, the cognitive style reflected in MT may manifest in everyday superstitious beliefs about lucky charms, or omens e.g. feathers, black cats. Historically, MT may have underpinned rituals associated with witchcraft, the public fear of which led to events such as the Salem trials (9). Even today, a million people across 95 countries believe in the power of similar practices (10), with recent UK census data highlighting a surge in related pagan religions such as wicca (11). While TAF may be expressed in contemporary popular culture from beliefs in paranormal concepts such as telekinesis, to New Age movement practices focusing on “manifestation”, many orthodox religions are intertwined with teachings of otherworldly phenomena that encourage MT. For example, the Bible explains that the power imbued in the Apostle Paul to perform healing miracles transferred into handkerchiefs and aprons that had touched him (12). The act of prayer may also be considered to exhibit parallels with other manifestations of MT intended to ward off harm, given the anticipated impact of the prayer intention to positively influence reality through the powers of God. The influence of culture on MT is emphasised (13), such that instead of prayer, similar forms of MT may manifest as e.g. spell casting, in cultures that follow alternative religions or hold different perceptions about supernatural entities.

Evidence for the inherent nature of MT can be drawn from the observation that it seems to be a normal part of childhood between 2–7 years of age. Young children see the world through a ‘magical lens’ and believe in the power of wishing, magic tricks, and monsters under the bed vanishing in response to the switching on of a night light. While MT in children can be associated with anxiety (14), it can also encourage creativity (15). It is therefore likely to reflect unbridled imagination at a time when children are beginning to understand symbolic representation and are yet to develop concrete operational or logical reasoning (16–18). Though MT seems to reduce with age, some related phenomena may persist into adulthood. These could include apophenia (perceiving meaning within randomness e.g. (19)), anthropomorphising (projecting human characteristics or mental states onto non-sentient objects or entities e.g. (20)), and perhaps even imaginary friends (21).

Given that MT seems to exist as a natural part of human development, and is not always problematic, its wider functions and psychological impact are worthy of exploration. An earlier review of the literature related to MT (22) discussed its role in OCD and psychosis, as well as highlighting early explorations in relation to anxiety, mood and eating disorders, whereby relationships with some of the latter conditions were less well established. Many questions remain. For example, what are the social and biological factors that encourage MT, and in which cases do related thoughts and behaviours become clinically relevant? Therefore the aim of this current systematic review was to incorporate the last couple of decades of research investigating MT, including TAF and MI, across the disciplines of psychiatry and psychology, to determine (1): Which populations have been studied and which measures have been (most) used when studying MT (2); Which demographic or environmental variables have been identified as likely predictors of MT; (3) The psychological (cognitive or emotional) correlates of MT; (4) The likely neurobiological underpinnings of MT; (5) Clinical implications that can be drawn from identified relationships between MT and psychiatric symptoms; (6) The main limitations associated with previous studies and questions to be addressed by future research.

## 2 Methods

### 2.1 Search strategy and data sources

A Web of Science core search conducted in January 2025, for articles in English, with no restricted date range containing within the abstract the search terms ‘thought action fusion’ OR ‘magical thinking’ AND ‘obsessive compulsive’ OR ‘schizo*’ OR ‘psychiatry’ OR ‘psycho*’ OR ‘personality’, yielded 427 records. Abstracts were first screened to ensure studies explored MT (referred to MT, TAF or MI) as typically defined and referred to psychiatric populations and/or psychological assessments. Duplicates and studies within the fields of marketing, philosophy/ethics or education were removed. The text of the remaining articles was then examined and the second round of exclusions (See **Supplementary Figure 1**) comprised papers that presented no new original data about MT, such as review or theory papers, new measure validation studies, or studies that used the term MT in a loosely descriptive way without including formal assessments (i.e. a standardised scale) to collect data about MT. Examples of the latter type of exclusions included case studies, qualitative studies, diagnostic interviews, or studies using spirituality/personality scales that didn’t capture specific MT data.

### 2.2 Study categorisation

After exclusions, the remaining 191 studies included in this review were organised into 11 secondary topics based on specific psychiatric disorders or other psychological factors (**Table 1**). A few papers spanned multiple topics (e.g. anxiety disorders and OCD), and so were categorised based on the lesser researched topic to ensure visibility of the full-range of topics. Where studies looked at traits associated with OCD or SSDs in a non-clinical sample, these usually applied standardised clinical and personality scales and are therefore categorised according to the clinical condition. In addition to key findings, **Supplementary Table 1** provides the complete list of reviewed studies (including those not cited herein), and presents the measures used by each study to assess MT as well as sample characteristics, organised by secondary topic. TAF and MI are used where applicable, with MT being used where applied by the scale developer or to refer to a combination of different measures collectively. Of the 191 studies in the current review, 28 were published prior to the previously identified review of studies on MT (22), but only 14 were cited therein.

**Table 1:** Focus of studies investigating magical thinking (inc. TAF).

Type	Topic	Number
Clinical	Obsessive compulsive disorder/traits	71
	Schizophrenia spectrum disorders/traits	45
	Anxiety or mood disorders	21
	Eating disorders	5
	Gambling compulsions	3
	Borderline personality traits/disorder	2
Non-clinical	Spirituality/religion	23
	Cognitive factors	10
	Early trauma	5
	Neurophysiological correlates	4
	Developmental research	2

### 2.3 Quality appraisal

A quality appraisal was carried out to help address aim 6, based on criteria that could be applied across all reviewed studies given the range of designs and methodologies. The first two criteria applied to how well the sample was characterised in terms of age, gender, ethnicity and any other informative data points, as well as its size. The third concerned how effectively the relevant MT data were presented, including subscale detail. The last two criteria fell under the quality of measurement, based on inclusion of measures for psychiatric symptoms, and additional MT measures, including whether these were self report or of a more experimental nature (**Table 2**).

**Table 2:** Study quality appraisal findings.

Evaluation criterion	Overall findings*
1. Sample characterisation and demographics (age ranges, genders, nationality, ethnicity, education etc.)	• A few studies only reported age ranges, especially for school samples. Mean ages are almost all from late teens to early thirties (fewer than 20% of studies have a mean age over 35). NC studies tended to be younger given many involved students. Child samples were more common under the OCD, SSD and ANX/MD topics, with a few studies on adolescents under ED. • Usually (71%) considerably more females, with just 20% of studies containing a roughly equal split (i.e. ~ 45 versus 55%) and only 9% with 60% or more males. NP, DEV and TRAUMA related studies were most balanced in terms of gender. NC studies usually had a higher proportion of females unless under the latter topics. • Ethnicity recorded in 30% of studies. Most student studies (NC) did not report ethnicity, and a few nationality only. Of the 57 studies that did include ethnicity data, 37 reported that about 66% or more of the sample or more were white/Caucasian, in fitting with the geographical spread (see section 3). A cluster of studies recruited Turkish or Korean participants, just a few samples from Greece, India and Singapore, and one study a large Arabic sample. NC studies under SPR/RELIG or COG most often reported ethnicity. • Around 50% of studies provided more data to better characterise their sample. Most commonly reported by this subset (91 studies) were education level or occupation (38%; 32%), religion (29%), marital status (28%) and socio-economic status (18%). Just a handful of studies reported variables such as IQ, family setting or urbancity. Additional sample characteristics were more often reported in CL than by NC studies, which often recruited students. • Religion was explored under SPR/RELIG, and a few studies recruited either exclusively Christian, Muslim or Hindu participants. The greatest number of studies represented a mix with Christianity (both Protestants and Catholics) or Islam being most represented, very few studies included sizable groups of Jews, Hindus or Buddhists.
2. Sample size	• 12.6% of studies had up to 50 participants, 25% 50 to 100, 18.3% between 101 and 200, and 44% of studies had more than 200 participants. • Most of the smaller samples were NP studies and most larger samples explored SSDs, and/or OCD from large community pools. While there were fewer studies in the DEV and GAMB categories, these samples were sizable. The smaller sample sizes were found in clinical OCD studies, and ED studies (often multiple small subgroups).
3. Administration of measures and presentation of related data i.e. use of subscales rather than total score or composite scores, providing statistics for comparison to other studies	• Mean and SD for the MT scale was clearly present in 70% of papers (usually within a table), with a further 7% of studies presenting additional information about the distributions of scores (medians, ranges, skewness, kurtosis etc.). A small proportion (6%) of articles reported counts or frequencies instead of parametric statistics (e.g. proportion of sample endorsing YBOCS MT items), and just a couple of studies presented median value only. The remainder did not report scale scores (often SPR/RELIG studies) although the data was incorporated into regression findings (10%). • Including data on individual subscales was relevant depending on the measure used, but applicable to the TAFS which was used by 101 studies (least frequently under the SSDs topic). Of this subset, 39% reported values for both TAF-M and TAF-L subscales, with reporting of TAF-LO and TAF-LS being a bit less common (36%) but more frequent for OCD studies. 20% reported only the TAFS total score and just a handful (5%) used the TAF-L or TAF-M subscale (just SPR/RELIG) alone.
4. Mental health screening or inclusion of scales to assess psychiatric symptoms	• Nearly half of studies (47%) included a combination of scales intended to measure symptoms of OCD, anxiety, depression and/or SSDs, and less commonly, measures for personality disorders or tic disorder. • Approx 20% studies included just OCD scales; 17% included no mental health measures; 5% included only a measure related to SSDs; and a few included only measures related to anxiety (2%) or depression (2%). OCD studies rarely included a measure of SSDs, and vice versa. • 7% of studies performed a mental health screen without including scale data. NC (SPR/RELIG, COG, NP) studies were least likely to include a psychiatric symptom scale or mental health screen.
5) Inclusion of multiple MT measures (self report or experimental)	• 49 studies used multiple MT measures. This was most common for the OCD topic but less so for other clinical disorders (e.g. SSDs, ANX/MD). • The more common combinations were TAF plus a self-report measure for OCD such as the YBOCS or OBQ, or occasionally both the TAFS and MIS. • 26 studies included a concurrent experimental measure of TAF, typically thought induction based on sentence cues. For a few of these (n=6), no results were reported (the task was used for fMRI). Results were more often presented for CL and then NC OCD studies, with a few for COG studies.

ANX/MD, Anxiety or mood disorders topic; CL, clinical studies; COG, cognitive factors studies topic; DEV; Developmental studies topic; GAMB, Gambling studies topic; MIS, Magical Ideation Scale; MT, Magical thinking; NC, Non clinical studies; NP, Neurophysiological studies topic; OBQ, Obsessive Beliefs Questionnaire; OCD, Obsessive Compulsive Disorder/traits (topic); SPR/RELIG, Spirituality and religion studies topic; SSDs, Schizophrenia Spectrum Disorders/traits topic; TAF, Thought Action Fusion; TAFS, Thought Action Fusion Scale; TAFS-L, TAFS likelihood subscale; TAFS-LO, TAFS likelihood other subscale; TAFS-LS, TAFS likelihood self subscale; TAFS-M, TAFS moral subscale; YBOCS, Yale Brown Obsessive Compulsive Scale. *See **Supplementary Tables 2**, **3** for further detail.

## 3 Results

### 3.1 Summary of study characteristics

Of the 191 studies (**Supplementary Table 1**), the most common measures used were the Thought-Action Fusion Scale ((2) TAFS; n=101), Magical Ideation Scale ((5) MIS; n=26) and Schizotypal Personality Questionnaire ((23); SPQ; n=26). The latter has a subscale (MT/odd beliefs) with items that fall under the definition of MI, and which was more often used for studies under the SSDs topic. Twenty-five studies (most investigating OCD/traits or cognitive factors) used some form of TAF or intrusive thought induction paradigm. This typically involved asking participants to write down a sentence that would relate to an unpleasant intrusive thought, and then rating responses e.g. anxiety ratings, neutralisation behaviours. Sample populations were most often from a university pool only (n=60), although sometimes screened for specific attributes (e.g. religion), with the second most popular population being clinically identified participants with a diagnosis of OCD (n= 46). Despite the high number of studies exploring the relationship between MT and SSDs/traits, few used clinical sources to recruit patients (n= 8), with most conducted in non-clinical university samples (n=17). Only eighteen studies specifically recruited children or adolescents, and most often under the OCD/traits, SSDs/traits and anxiety/mood disorders topics, with a couple of adolescent studies in eating disorders (EDs). The majority of studies were conducted in the USA (30%), Australia (10%), UK (10%) or other European countries (20%). Otherwise, a notable amount were Turkish (8%) or South Korean (5%) samples, with around 10% of studies recruiting internationally (further discussion in 4.1).

### 3.2 Summary of quality appraisal findings

Quality appraisal highlighted both strengths and weaknesses of the studies, some of which were more apparent for certain topics or in relation to clinical or non-clinical sources. Findings are presented in **Table 2** with further detail available in **Supplementary Tables 2**, **3**. Strengths often included sample size, and frequent presentation of scale data within individual subscales where applicable. The most common weaknesses included limited characterisation of the sample beyond age and gender, and a lack of inclusion of concurrent MT measures (especially of a more objective nature), and/or psychiatric scales or mental health screening. The quality appraisal process highlighted a range of limitations and explicit pointers for future research (see 4.6).

### 3.3 Clinical topics

#### 3.3.1 Obsessive compulsive disorder and traits

Overall, the evidence demonstrates the clear cross-cultural relevance of TAF (e.g. (24, 25)) with TAF-L being a better indicator of OCD or milder symptoms: OCS. However, some studies exploring these traits in community samples highlight an effect of culture or nationality (e.g. (26, 27)). Importantly, findings may vary according to whether TAF or MI is assessed. For example, TAFS and MIS scores show opposite patterns across Icelandic versus Australian students (28). Other demographic influences include possible age effects, and while MT levels are not necessarily linked to OCD severity in children (29), higher MT has been found in severe childhood OCD (30). Interestingly, older children with OCD exhibit more MT than younger children (31) which may suggest atypical development. Maternal TAF and metacognition are strongly linked to 7–11 year old children’s thought suppression and TAF-M respectively, but this pattern isn’t found in adolescents (32). Correlations between MT, metacognition and problem solving in studies involving children (33) suggest that MT is linked to the handling of challenging situations. In adults, TAF affects inferential thinking (34), such that high TAF is associated with making decisions based more on feelings of certainty or likelihood versus more objective factors (35). One study found that TAF-M specifically was positively linked to self-certainty and negatively to self-reflectiveness (36). Further research is needed to understand the relationships between different forms of MT, cognitive development, and the symptoms of OCD.

Student studies have explored the relationship between TAF and perceptions of mental contamination (e.g. (37)). Both core disgust elicited by potential physical contaminants such as bodily fluids, and moral disgust in response to physically or psychologically harmful behaviours, can increase TAF (38). Mental contamination concerns mirror perceptions of immorality and indecency (39) and involve feeling ‘dirty’ and needing to neutralise the sensation (40). In OCD samples, mental contamination is correlated with TAF (41). TAF induction can increase shame (42) and TAF-M specifically is related to high scrupulosity (i.e. excessive guilt or anxiety about moral issues) in OCD (43). Interestingly, when rating feelings towards sentences designed to elicit TAF, reaction times of controls are related to TAFS scores, while reaction times of those with OCD are related to guilt measures (44). Many studies have revealed the association between TAF and measures of responsibility ((45); TAF-M specifically: (46); TAF-L specifically: (37); in students (6)) or thought suppression, whereby both TAF and thought suppression contribute to OCS (46–49). Collectively, these studies highlight the importance of moral emotions in combination with MT as playing a key role in OCD.

While research in community samples shows TAF-L is related to frequency of obsessive intrusive thoughts and behaviours (50, 51), one study found that TAF-LS (TAF L about the self) was less closely associated with OCD than TAF-LO (TAF-L about others) and TAF-M (52). Another study in students found that object checking was specifically related to TAF-LS whereas TAF-LO was more closely related to generalised anxiety and interpersonal checking (53). However, clinical studies suggest that high TAF doesn’t necessarily predict disability level (54) or track with symptom reduction over time (55), even though numerous studies report changes in TAF after successful interventions (e.g. (56)). A poor response to CBT has been predicted by high MIS scores (57) or high TAF-M (58), and good outcomes may be predicted by lower baseline TAF (59). However, findings can be influenced by the OCD measures used i.e. TAFS may be less closely related to Obsessive Compulsive Inventory-Revised (OCI-R: (60)) than Dimensional Obsessive Compulsive Scale (DOCS: (61)) scores (62). These studies emphasize the importance of measure selection and application (see also **Table 2**).

Non-clinical studies exploring OCS have highlighted negative affect as a potential partial mediator of the relationship between these traits and both TAF and MI (4). However, MT is not always associated with depression in student samples (47), and TAF-L can be prominent after controlling for negative affect (63). One study in young people controlled for depression and found high TAF is associated with compulsions and superstition in teenagers recruited from the community (64), while another found increased MT and related obsessions in youth were associated with hoarding (65, 66). Indeed, MIS scores specifically have been linked to hoarding in adults (67), as well as autistic symptoms and aggressive obsessions (68). MI may therefore be a useful indicator of psychiatric vulnerability in young people.

TAF induction has been used in a number of studies to expose the neurological correlates of MT and related symptoms of OCD. In a non-clinical sample, when compared to positively valenced TAF, negatively valenced TAF recruits additional regions thought to be relevant for salience and self-referential processing, such as the insula, medial prefrontal cortex, precuneus and thalamus, with precuneus activity also being related to DOCS (61) scores (69). Similarly, TAF induction is associated with lower power within the salience network in people with OCD versus controls (70), and another study found both higher TAF and greater precuneus activity in OCD compared to participants with low level traits or OCS (71). TAF scores can also covary with functional connectivity between the mid-cingulate and insula in OCD (72), and precuneus connectivity covaries with change in TAF scores in patients undergoing therapy OCD (73). These findings are discussed further in section 4.4.

#### 3.3.2 Schizophrenia spectrum disorder traits and symptoms

Studies considering SSDs/traits found that in addition to female gender (74–77), environmental variables such as urbanicity (78, 79) can be correlated with MT. Idiopathic Environmental Intolerance i.e. the experience of somatic symptoms attributed to environmental agents, has also been linked to MI (80), but whether this is due to other sensory or cognitive abnormalities that characterise SSDs is unclear. Atypical sensory effects associated with MI and related measures of ‘odd beliefs’ include poor performance on somatosensory signal discrimination tasks (81) in first degree relatives of people with schizophrenia and on a multisensory temporal processing task in healthy students high in schizotypal traits (82). Therefore, further research may confirm sensory disturbance as contributing to both SSDs and MT in general.

The relationship between MT and beliefs about conspiracy may reflect a common underlying factor in the form of agency attribution. Indeed, TAF is strongly correlated with sense of agency (83). The role of intention attribution in MT raises the likelihood of an association with social cognition. While very few studies have explored this possible relationship, one that did reported a positive correlation between TAF and emotional reactivity towards others (83). Other studies investigating relationships with interpersonal functioning have found that MT is less predictive than other schizotypal traits (84). One study exploring links between childhood abuse (see also 3.4.3) and schizotypy concluded that MT was associated with core schema negative-other beliefs (85). Social factors are also highlighted in the negative association between MI and loneliness, as found in a community sample (86), and where aspects of positive schizotypy such as pseudoscientific beliefs increase during isolation (87). Their potential importance is explored further in 4.3.

In relation to cognitive and perceptual functions, high MIS scores are associated with hypnotisability (88), false memory and perceptual aberration (89). Indeed, fantasy proneness is suggested to be a key link between TAF and schizotypy (90). Other studies have linked MT to creativity (e.g. (91, 92)) and more specifically, MI to aspects of creative experience such as ‘positive flow’ (93). Neurophysiological studies of MI in association with schizotypal features have also documented unusual cognitive perceptual findings e.g. altered visual evoked potentials towards neutral stimuli (94) and atypical visuospatial processing in association with high MI (95). Similar effects were found in both non-clinical and mixed clinical populations in relation to delusions (Bipolar disorder and SSDs: (96)). Taken together, these studies emphasise a role for perceptual changes and creative imagination as central to MT and related symptoms of SSDs.

When using measures of schizotypy, MT is common in OCD samples (97), with a prevalence of 43-64% in healthy adolescents or students (98, 99). TAF-L is high in non-clinical samples in association with schizotypal characteristics (100) after controlling for OCD and mood disorder, and more specifically, TAF-LO is higher in schizophrenia (101). In a study of people at risk for psychosis, SPQ items related to MI were amongst the best predictors of transition (102). The MIS can also discriminate well between patients with OCD or psychoses with or without auditory hallucinations (8). Non-clinical studies have linked high MI to persecutory ideas (103, 104), ideas of reference (105), and hoarding tendencies [(106); SPQ]. However high MT scores on various measures in samples without clinical psychosis (**Supplementary Table 1**) are unrelated to suicide risk (107), perceived stress (108), depression (109), or functional difficulties (110).

Using a twin study, Torgersen et al. (111) showed that MT was part of the genetic core of schizotypal personality. Specific genetic markers were associated with higher intensity of MI in one nonclinical sample (112), and another study showed polygenic risk score for schizophrenia predicted higher MT in adults without psychosis, without the decrease with age seen in people with a low risk score (113). A potential role for neurochemistry was suggested by a study that found a ketogenic diet was associated with reduced MI over time (114), while another study reported a specific correlation between MT and the size of the right fusiform gyrus (115) in a non-clinical sample with schizotypal traits. There is a stark lack of neuroimaging data pertaining to MT in clinical samples with SSDs/traits and relatively little has been revealed in terms of biological factors.

#### 3.3.3 Anxiety and mood disorders

Findings of the numerous studies comparing OCD to mood and/or anxiety disorders have concluded that high TAF in OCD is associated with negative affect e.g. (116). MI can also be associated with depressive symptoms in non-clinical samples (117). However, in some studies OCD patients don’t exhibit higher TAF (118, 119), or specifically higher TAF-M than found in major depression (120). This may reflect self-blame and ruminative guilt in the latter, given that during TAF induction, patients with major depression activate inferior frontal regions, insular gyri and caudate more than controls, and activity co-varies with both TAFS scores and rumination (121). Another study using more fine-grained analysis, revealed that compared to healthy controls, depressed participants had higher total, uncontrollable and self-suicidal TAF subscale scores, but lower positive controllable TAF (122). These findings highlight how emotional valence may interact with cognitive bias to determine the pattern of MT in terms of the kinds of intrusive thoughts and obsessions and suggest MT and depressive rumination may share common neural underpinnings.

While depression is commonly correlated with high TAF (sometimes TAF-M in particular: (120)), the pattern in anxiety disorders appears less predictable. Research involving clinical samples sometimes indicates that MT or TAF specifically reflects OCD rather than depressive or anxiety disorders (e.g. (116, 123, 124)), but on the whole findings are rather mixed (see **Supplementary Table 1**), perhaps because anxiety disorders constitute a more heterogenous group. A few studies report higher TAF-L in generalised anxiety disorder (GAD) versus OCD e.g. (125)), or that GAD duration is positively associated with MI (126). Regardless of differing severity, TAF does seem to contribute to intrusive thoughts in specific anxiety disorders such as illness anxiety disorder (127), and treatment can lead to a reduction in TAF in both OCD and anxiety disorders (128). MI can also be high in patients diagnosed with somatoform disorders and health related anxiety when controlling for mood (129), again highlighting a possible link with sensory dysfunction.

#### 3.3.4 Eating disorders

Thought-shape fusion (TSF) is a variant of TAF whereby thinking about eating certain types of food increases a person’s estimate of their shape and/or weight, elicits a perception of moral wrongdoing, and/or makes the person feel fat. Radomsky et al. (130) found that those participants with anorexia nervosa who experienced such thoughts felt the need to perform neutralisation and exhibited higher TAFS scores. In relation to Bulimia, whether symptoms centre more on binging or purging seems to be predictive of TAFS subscale scores (131). In general, higher TAF in EDs is associated with greater symptom severity including more obsessional thoughts (132) and greater emotional disturbances (TAF-M specifically: (133)). One additional study explored ED pathology in relation to OCD and MT in an adolescent school population (134) and found that found that TSF was a better predictor of depression than TAF.

#### 3.3.5 Gambling compulsions

Three papers investigated MT in relation to gambling compulsions. Passanisi et al. (135) found a positive association between MIS scores and tendency to gamble in a healthy adolescent sample, in terms of illogical or risky decision making e.g. gambler’s fallacy. However, another study (136) that recruited healthy twins, reported that not all gamblers evidence MT on the MIS, and that MT tended to cluster with negative emotionality and more problematic or non-strategic gambling activities. Similar conclusions were drawn by Teed et al. (137), who found that non-strategic gambling was more closely linked to MT in the form of beliefs about luck. The students more likely to exhibit problematic gambling were also more likely to believe in good fortune being transferred through objects.

#### 3.3.6 Personality disorders

Just a couple of studies have explored MT in relation to Borderline Personality Disorder (BPD). The first showed that after excluding those with SSDs, diagnostic interviews involving participants with BPD showed more evidence of MI e.g. beliefs about telepathy, clairvoyance and superstitious beliefs, compared to other personality disorders (138). Another study involving participants with borderline traits plus mood disorder found that more MT as measured by the Trait Character Inventory (139) predicted dropout of a CBT group focusing on emotion management (140).

### 3.4 Non-clinical topics

#### 3.4.1 Spirituality and religion

The majority of studies explored MT in terms of TAF, in samples including Christians or Muslims, comparing religious and/or non-religious groups. For example, Berman et al. (141) found that TAF induction led to higher TAF-M and neutralisation behaviours in Christians versus agnostics/atheists. Some studies report differences between Protestants versus Catholics in terms of TAF-M (142) but others find no difference (143). Lutheran pastors affiliated with more conservative denominations evidence higher TAF-M than less conservative pastors (144), and other studies have found higher TAF-M in Christians (145) or Muslims (146) compared to Jews. Another study reported high TAF in Muslims regardless of level of religiosity, while there was a positive association between these variables in Christians (147). In relation to cultural influences, one investigation that re-examined data collected at various times since 1928 (148) revealed intriguing relationships between practices related to MT (e.g. counting objects, lucky objects, other superstitious rituals) and variables including religiosity. Country of origin played a role in the use of magic, how this was practiced, reasons for its application, and its continued use over time. Interestingly, more religious than non-religious participants reported practicing magic, with the highest number in India, followed by Germany and then South Korea. However, it is unclear whether MT was the casual factor in this association.

Research in student samples has also revealed relationships between religious belief and TAF, in TAF-M specifically (149), with a couple of studies emphasising relationships between Christianity and perceptions about the immorality of intentional negative thoughts (150, 151). More broadly, MT is correlated with aspects of spirituality considered to be important for general well-being, such as connectedness (152) and intolerance of uncertainty (153). The relationship between religiosity and TAF-M has been found to be mediated by both universality (the belief that the universe is ordered and humanity is connected) and moral vitalism i.e. beliefs in the influence of good and evil forces (154, 155, respectively). Aside of specific religious faith, MT has been found to positively correlate with prayer (156) and mystical experiences (157), as well as mind-body therapy use (158).

While TAF-M has been found to mediate the association between OCS and religiosity (e.g. (159)), TAF-L mediates the relationship between OCS and paranormal beliefs (160). Furthermore, while religiosity is positively related to TAF-M, it is negatively related to TAF-L, at least in Protestant Christians (142). The finding that the symptoms of individuals with OCD can be negatively associated with spirituality (154) could reflect similar associations. However, specific beliefs about God and worries related to punishment can further influence effects related to MT and OCS (161, 162). These studies highlight the potential for inverse relationships between MT related to religion/spirituality and psychopathological symptoms in terms of OCD, and that spiritual characteristics may moderate relationships between MT and psychiatric symptoms.

#### 3.4.2 Cognitive factors

Studies exploring cognitive factors suggest that vulnerability markers for high MI may include ethnic identity and related religiosity (163), as well as psychological mindedness (164). The latter concept was found to be inversely related to MT and refers to using thoughts, feelings and behaviours in order to understand the self and others. Links between stress and increased MT appear more likely in those with lower ambiguity intolerance (165), and attachment anxiety may specifically encourage TAF within relationship-related OCD (166). While perceptions of inflated responsibility are also correlated with TAF (167), there could be cultural variations (e.g. (168)).

From a therapeutic perspective, exploring the idea of belief controllability can reduce TAF (169) and related anxiety or neutralisation (170) after induction of intrusive thoughts in non-clinical samples. Increasing cognitive dissonance about TAF beliefs may help further (171), although some studies find effectiveness of methods vary according to TAF subtype and may not affect judgments about thought intrusiveness (172).

#### 3.4.3 Early trauma

There is emerging evidence that childhood maltreatment could encourage MT. A couple of studies suggest that emotional abuse in particular, may be predictive of high TAF-L (173, 174), but others have found broader associations. For example, one Chinese study found that MT was positively associated with a range of forms of abuse (physical, emotional, sexual) as well as physical neglect, although MT and emotional neglect were found to be negatively correlated (175). Another study revealed a trend between the cognitive perceptual factor of SPD (which includes MI) and severe sexual abuse (176). Finally, a Turkish study explored similar relationships in adults with OCD (177) and didn’t find a relationship between TAFS scores and childhood trauma, although TAFS scores were correlated with dissociative experiences.

#### 3.4.4 Neurophysiological correlates

So far, neurophysiological studies in non-clinical samples (**Table 1**) have documented relationships between TAF induction and precuneus activity (178) and between MI and reduced left hemisphere dominance (179). Other findings include an association between MT and activity in frontal regions identified using electroencephalography (180) and transcranial magnetic stimulation (19). Studies employing brain imaging that focus on specific clinical groups are found elsewhere in this review (e.g. 3.3.1). These findings are considered further in section 4.4.

#### 3.4.5 Developmental research

While developmental research is perhaps surprisingly scarce, a couple of studies have explored MT in typically developing children, acknowledging that this phenomenon arises as a part of typical cognitive development between 2 and 6 years of age. Bolton et al. (181) found that there could be associations with obsessions and compulsions, although more often with anxiety in boys specifically. The developmental trajectory was not found to be linear in that study, while another study reported that it tended to decline with age (182), and that beliefs in e.g. the powers of wishing, were closely linked to compulsive rituals in children. Other studies involving young populations focused on relationships with OCS/OCD (see section: 3.3.1).

## 4 Discussion

### 4.1 Populations and measures used when investigating MT

After the TAFS, the MIS and SPQ (odd beliefs/MT subscale) were the next most commonly applied measures. The TAFS, and TAF induction, was used less often for SSDs/traits studies, perhaps because MI or MT in general is thought to be associated with SSDs but TAF moreso with OCD. Studies investigating SSDs/traits rarely benefitted from a concurrent measure of MT, unlike those exploring OCD/traits. However, the vast majority of non-clinical studies (usually concerning spirituality/religion), used measures of TAF rather than MI. Given that MT measures were self report, the associated weaknesses are applicable to the large amount of studies that didn’t include related experimental measures, such that the knowledge base is likely to be most reliable for OCD related studies (although the relationship between TAF and various measures of OCD symptoms can vary e.g. (62)). There is also the more general difficulty in establishing causal relationships given the reliance of studies on correlational methods.

Despite the breadth of studies and range of populations sampled, there is a stark lack of data involving participants from Africa or Asia. Most lacking are studies involving older, non-white males, particularly from minority religious groups/denominations. In terms of clinical samples, despite the number of studies related to SSDs, relatively few recruited from clinical sources, and very few studies were found in other relevant clinical groups such as EDs, personality disorders or neurodevelopmental disorders e.g. autism, Tourette syndrome. Only eighteen studies reported specific inclusion of children or adolescents, and such studies were more common under the OCD/traits, SSDs/traits and anxiety/mood disorders topics, with a couple of adolescent studies under EDs.

### 4.2 Demographic and environmental variables associated with MT

A couple of studies have shown that MT tends to decrease with age in typically developing children, although further research is needed to confirm its relationship with other aspects of cognitive development, and how typical patterns may vary in relation to the later onset of various psychopathologies. There could also be interactions with gender, whereby in general MT could be higher in females (74, 76, 77), although this requires further confirmation. In relation to family environment and relationships, maternal TAF can influence younger children (32), and the associations shown between MT and trauma could highlight a potential influence of attachment (see also 4.3). Environmental influences include factors linked to urbanicity (79), with additional research needed to specify further.

The topic providing most supporting evidence in the non-clinical domain is the connection between MT and religion, and perhaps with TAF-M in particular. Relationships between TAF, religiosity and OCS appear common across different faiths, although finer points vary according to specific religious beliefs and perceptions about God. MT also manifests in slightly different forms across cultures, with one example being the practice of magic in countries such as India (148). Therefore, many of the reviewed studies highlight the importance of cultural considerations within clinical practice (183, 184). These studies, and indeed non-clinical research overall, also emphasises an important link between perceived agency, and the meaning of actions in terms of ethics and morality. These will be discussed in the following section in terms of relationships between cognition, spirituality and MT.

### 4.3 Relationships between psychological (cognitive or emotional) variables and MT

Cognitive factors likely to be associated with TAF in non-clinical samples include perceptions of responsibility (as also found in numerous clinical samples) and sense of agency. These aspects may comprise the common link between SSDs and social cognition in the form of attributions of agency and intention. Need for cognition, creativity, and fantasy are also positively related to MT, and perhaps MI more specifically (94), whereas self-reflection can be negatively related to TAF-M (35). Such findings could imply a benefit for future studies to explore links between the cognitive traits associated with MT and dopamine, as well as convergent and divergent thinking styles. TAF-L in particular, seems likely to reflect faulty probabilistic reasoning, given that previous studies imply reasoning based on feelings about likelihood as opposed to more logical contextual information (34), although this requires further investigation.

A few clinical studies involving patients with OCD explored relationships between moral disgust and intrusive thoughts as captured by the TAF-M subscale, measures of perceived contamination, and responsibility or religiosity (e.g. (38, 160)). Their findings emphasise parallels between perceptions of mental and physical contamination, which could be expressed as symptoms of OCD (e.g. concerns about germs), or of SSDs (e.g. concerns about thought control). These studies illustrate how TAF specifically, can be seen as the physical materialisation of mental states such as intentions or desires, as manifest within external actions or events. The moral implications of mental and physical contamination may also feed into religious teachings and practices such as ritualistic washing or baptism, while transmission of likelihood materialises as the superstitious use of lucky objects. Outside of certain socio-cultural contexts, similar beliefs have potential to be viewed as pathological, especially where they may impair daily function or wellbeing (183, 184).

The role of mental states (beliefs, intentions) in MT highlights the potential importance of social cognition, which often involves identifying the mental state underpinning an action or remark. The relationship between MT and social cognition remains largely unexplored, aside of a couple of relevant findings in non-clinical studies highlighted by this review. Firstly, there is the inverse relationship found between psychological mindedness, which involves reasoning about mental states, and TAF (164). Secondly, there is evidence of positive correlations between TAF and aspects of empathy such as personal distress and negative correlations with perspective taking, which suggests those high in TAF experience more negative emotions when around others who are in distress (83). The pattern implies TAF may be positively associated with aspects of empathy, but perhaps negatively related to more abstract forms of social reasoning. It may be speculated that a positive relationship between TAF and relying on non-verbal cues rather than of hypothetical reasoning about perspectives could help to explain why clinical groups associated with high TAF demonstrate certain kinds of bias on social cognitive tasks. This suggestion is supported by findings that patients with Tourette syndrome or schizophrenia tend to assume that actions directly reflect intentions (185), demonstrating a kind of ‘action-thought fusion’ when explaining the reasons for ambiguous social behaviours such as faux pas (186), and may even perceive intentions reflected in random movement (187, 188).

The relevance of emotion to MT is highlighted in clinical studies involving mood disorders and more generally in TAF induction studies that demonstrate the role of shame or guilt in intrusive thoughts. Such emotions are highly relevant to the experience of abuse, which can encourage the likelihood of certain personality traits, as well as problematic emotional responses towards others (85, 166). TAF exhibits positive associations with attachment anxiety (166) and trauma, and anxious attachment has been highlighted as a contributory factor to the development of both OCD (189) and EDs (190). TAF-L is positively associated with OCD and SSDs but inversely related to religiosity and OCD (142, 154), and Christianity may moderate relationships between MT and psychiatric symptoms (151). Therefore, perhaps spirituality or religion can provide a beneficial outlet for MT, or even encourage the kind of protective emotional connection or secure attachment (e.g. with God), that the MT observed in numerous mental health disorders may signal a need for. In relation to MI, a couple of studies hint at a positive correlation with loneliness and/or social isolation (86, 87). If tendencies to attribute intentionality in a magical way become more likely where social connections are compromised, MT may function to provide meaning and connectedness (152) between the self and the external world. That is, MT involving the identification of agents or entities, could sometimes be supportive to mental health. In such ways, MT could encourage resilience in the face of psychological stress, as perhaps hinted at by the reported association between MT and problem solving (36), and associated skills such as creativity and flexibility. Further research is needed to confirm the meaning of relationships between TAF and social connectedness, especially in clinical samples, to explore the potential for MT to perform any beneficial function.

### 4.4 The neurobiological underpinnings of MT

In relation to the neural underpinnings of MT, the few non-clinical studies that are available highlight the precuneus as an area of interest (e.g. (178)). This sentiment is echoed by a couple of studies involving patients with OCD (e.g. (71)). Indeed, precuneus connectivity covaries with therapeutic reduction in TAF scores (73). These findings may reflect the role of the precuneus in making sense of external perceptions in relation to the self (191), and further implicate attribution of agency, which appears central to TAF (83). The precuneus has been highlighted in social cognitive studies using perspective taking tasks (e.g. (192)) and may underpin mental imagery involved in TAF. In fact, clinical groups that demonstrate high TAF and atypical intention attribution (i.e. Tourette syndrome; SSDs), exhibit atypical activity in brain areas associated with social cognition such as the precuneus and the temporo-parietal junction (193–195). These neuroimaging findings compel investigation of the potential relationships between MT, perspective taking and related neural circuitry.

One study highlighted reduced activity of the anterior cingulate (70) in association with TAF, and TAF scores have also been shown to be correlated with functional connectivity between the mid-cingulate and insula in OCD (72). The insula contributes to the realisation of internal visceral and affective states (196), as well as signalling salience along with the anterior cingulate (197), hence these regions likely subserve key cognitive and emotional functions that affect the attribution processes involved in TAF. Given that an imaging study of TAF induction found greater insular activation in major depression in association with both TAFS scores and rumination (121), and evidence of relationships between TAF, alexithymia, and interpersonal reactivity (83), awareness of internal states and related affect should be studied alongside social cognition in future studies of TAF.

While the neurochemical correlates of MT have not been formally investigated by studies included in this review, a few observations imply that a shared underlying basis between agency misattributions and mood changes could lie in dopamine, or neurochemical changes involving the reward system (186). The first is the role of dopamine in signalling salience and valence, given that MT typically involves attributions about highly significant events that are very unfortunate (or fortunate). Another is the likely link between MT and other forms of cognitive distortion seen in other conditions involving dopamine dysfunction, beyond SSDs. For example, one previous study showed that Tourette syndrome, which often involves OCS, can also feature the impulsive probabilistic reasoning bias referred to as jumping to conclusions (198), with another recent study reporting a similar pattern in early, cognitively intact, medicated Parkinson’s disease patients (199). Future research could explore the possible relationships between dopamine dysfunction and reasoning processes linked to MT, in movement disorders.

### 4.5 Potential clinical implications and relationships between MT and different types of psychiatric symptoms

The relationships identified between MT and specific kinds of psychiatric symptoms are summarised in **Table 3**. In terms of clinical diagnoses, OCD and SSDs seem to be most closely related to TAF-LO, with a less consistent relationship between OCD and TAF-M. Discrepant findings across studies in relation to anxiety disorder could reflect the challenges of differential diagnosis, while high TAF in both depression and OCD may share common underlying factors in the form of guilt, worry and obsessional or ruminative cognition. One study suggested that TAF subscales may differentiate between anxiety (correlated with TAF-L) and depressive (correlated with TAF-M) symptoms (200), but further research is needed to confirm this possibility. While TAF-L is associated with schizotypy in non-clinical studies (e.g. (100)), TAF-M specifically is greater in those with chronic schizophrenia versus controls (201) highlighting the importance of clinical sampling.

**Table 3:** Implications for future research and clinical practice.

Question/topic	Clinical implications	Research implications
Populations and measures used when investigating MT	• Limited scientific literature on MT in some cultures e.g. African, Asian, and how it is related to cultural norms and mental health • Knowledge base largely reliant on self report measures and assessment should be bolstered by objective observations • Causal relationships largely undetermined	• Studies exploring SSDs/traits in particular should use concurrent MT/MI/TAF measures • Future studies should sample older, non-white, males, minority religious groups/denominations • More rigorous experimentation required • Under-researched populations include disorders of personality, neurodevelopment and movement
Demographic or environmental variables associated with MT	• Influential demographics include age, gender, religious and cultural background. Relationships and trauma history may inform diagnosis and treatment planning • Environmental influences may include urbanicity, family setting and social connections • Cultural, religious, and spiritual findings emphasise the need to understand the potential functions and health implications of MT within broader context, as well as the importance of cultural formulation	• Developmental trajectory of MT is poorly characterised. Studies should explore MT during development alongside social cognition, creativity, imagination and phenomena such as anthropomorphising and apophenia • Longitudinal studies may address how normative MT diverges into psychopathology with age, identifying predictors of risk and potential resilience • Systematic inclusion of demographic and environmental variables is encouraged to elucidate relationships and allow for cross-comparison • MI has been less well explored than TAF in terms of relationships with religion/spirituality
Relationships between psychological (cognitive or emotional) variables and MT	• Religious beliefs or related practice could support a potentially adaptive output for TAF • Loneliness or low social connectedness could encourage MT • MT can signal attribution bias with wider clinical implications in terms of decision making based on emotion rather than logic or factual information e.g. impaired probabilistic reasoning could underlie both gambling tendencies and high MT • Negative self perceptions or related emotions such as shame or guilt could influence judgments about responsibility and agency, and in turn encourage TAF	• TAF-M specifically may be positively associated with moral cognition and ethical decision making but this is yet to be explored • Studies suggest a positive association between TAF and empathy but perhaps an inverse association with theoretical perspective taking. • MT could predict increased agency attribution (e.g. anthropomorphism) and atypical judgments about intention on social cognitive tasks • Studies should explore a number of different social cognitive tasks alongside measures of MT and alexithymia
The neurobiological underpinnings of MT	• Neurological damage (e.g. to precuneus) may influence MT but no case studies and findings are preliminary • Little understanding of the influence of pharmacological agents Possible associations with dopamine dysfunction or neurotransmitters implicated in mood disorder/OCD e.g. serotonin • A few studies report changes in morphology of sensory areas e.g. fusiform gyrus in association with schizotypal traits including MT • Preliminary research suggests that changes in functional connectivity measures coincide with positive treatment response and reduced TAF in OCD	• Tasks that isolate the various processes involved in MT (multiple forms of TAF such as self v other, positive and negative valence, etc.) are needed for imaging studies. • Very few imaging studies have been conducted in patient groups or in relation to SSD traits • Treatment trials (especially in OCD and SSDs) should include outcome measures linked to MT • The possibility that precuneus activity may underlie a visualization component to TAF while cingulate/insula activity underpins an affective component should be explored
Specific relationships between MT and different types of psychiatric symptoms	• Mood disorder (especially guilt and rumination) could be a risk factor for TAF • Difficulties in distinguishing between SSDs and OCDs (and perhaps milder mood/anxiety disorders) using MT scale scores emphasise the challenges of differential diagnosis • Trauma history could increase likelihood of certain personality traits and mental health problems (e.g. schizotypy), as well as other cognitive or emotional issues that interact with MT • Magical ideation may predict hoarding or aggressive obsessions. • Reasoning biases associated with MT may encourage jumping to conclusions, paranoia and superstition • Relationships with problem gambling suggest screening for impulsivity and compulsivity is helpful when MT is identified • Elevated magical ideation scores can signal increased likelihood of hallucinations and perhaps sensory symptoms	• MT levels could be a predictor of illness progression (SSDs) or treatment outcome (OCD) but patterns need to be established • It may be helpful for studies to explore relationships between specific symptoms and different forms of MT e.g. dissociation; depression; hoarding; perhaps in transdiagnostic studies • Studies should seek to confirm whether TAF subscales (or perhaps the ratio between subscale scores) may better predict specific symptoms or disorders e.g. TAF-M a better predictor of OCD; TAF LO higher in clinical versus non-clinical cases etc. • Research in transdiagnostic samples using concurrent measures of TAF versus MI could help tease apart clusters and validate assigned diagnoses • Studies are needed that explore the pattern of MT in relation to function and disease severity over time in order to identify mediating and risk factors

MI, Magical ideation; MT, Magical thinking; OCD, Obsessive Compulsive Disorder; SSDs, Schizophrenia Spectrum Disorders; TAF, Thought action fusion; TAFS, Thought Action Fusion Scale; TAFS-L, TAFS likelihood subscale; TAFS-LO, TAFS likelihood other subscale; TAFS-LS, TAFS likelihood self subscale; TAFS-M, TAFS moral subscale.

MT can be domain specific in EDs manifesting as thought shape fusion, while a pattern of more generalised MT in association with sensory and perceptual changes (e.g. (115)) is seen in association with schizotypal traits. Indeed, MI may exhibit closer associations with sensory and perceptual symptoms (8), as well as certain kinds of obsessions (**Table 3**). It is also worth noting any broader relationships between MT and sensory or motor functions, such as high MT in tennis players (in terms of superstitious behaviours: (189)). These findings emphasise the importance of further study in terms of the mind-body connection as reflected in TAF.

Correlations between hoarding and MT could be explained by mediating factors such as attachment disorder. There are too few studies to draw conclusions in relation to personality or neurodevelopmental disorders. However, childhood maltreatment is likely to be one transdiagnostic risk factor, as this seemed to increase the likelihood of MT. Dissociation could be a common link that further research may reveal to influence the development of both MT and psychosis. One student study showed that TAF-M mediated the association between OCS and religiosity, whereas TAF-L mediated the relationship between OCS and paranormal beliefs (158), which characterise MI. Such findings encourage more detailed examinations of the relationships between various forms or manifestations of MT and a range of psychiatric symptoms, in larger, transdiagnostic samples.

### 4.6 Limitations associated with reviewed studies and questions for future research

Quality appraisal (**Table 2**) highlighted that weaknesses in previous studies often included poor characterisation of the sample in terms of ethnicity, and this review highlights the importance of gathering data related to nationality/culture as well as religion and mental health status, to aid interpretation and related clinical implications (183, 184, 202, 203). For example, there are notable parallels between the risk-aversion rituals or neutralisation behaviours being performed in response to intrusive thoughts, and religious sacraments or ordinances (e.g. confession; baptism) as well as tradition related to ritualistic magic. Few studies applied clinical scale cut-offs or detail on scale data distributions although this would be useful to enable comparisons across studies. Future research should also aim to include multiple concurrent measures associated with MT to improve validity and not rely exclusively on self-report scales where possible. It was difficult to draw conclusions pertaining to more under-researched topics highlighted in this review, including the role of MT in addictive behaviour, as well as EDs and personality disorders. The lack of research involving autistic spectrum disorder is notable given the frequent symptom overlap with OCD and/or SSDs (e.g. (204, 205)). Furthermore, few qualitative studies were included, and it would be interesting to see if a future review focused on such studies would come to similar conclusions.

There are significant discrepancies in the findings related to certain topics, which may reflect variation in measures as well as individual sample characteristics. Studies investigating links between TAF, anxiety and depression in particular exhibit rather mixed findings, which may reflect symptom severity, or perhaps difficulties related to differential diagnosis. Studies including only university students may also fail to capture the more extreme ends of the spectrum in terms of MT and other clinical symptoms, so more replication involving clinically sourced samples would be helpful. Another problem is that in addition to search terms identifying only studies involving formal measures of MT, measurement instruments do not always map cleanly onto conceptual definitions. Finally, there are the more general limitations of reporting and publishing bias, as well as the limitations of self-report measures.

One specific area worthy of further study is the role of MT in cognitive development, particularly in terms of whether early atypical thinking patterns may constitute indicators of later psychiatric vulnerability. In relation to cognition more generally, little is known about how verbal skills, deductive reasoning or IQ, may be related to MT, or more specifically, subtypes of TAF. For example, TAF-L is likely related to probabilistic reasoning (e.g. jumping to conclusions), whereas TAF-M could be more closely connected to empathy. It is also the case that most studies consider TAF only in relation to negative effects. Controlling for valence could shed further light on the role of the reward system, and further elucidate relationships with gambling, and potentially also dopamine, and potentially, motor function. Tentative implications for future research and clinical practice are summarised in **Table 3**.

### 4.7 Conclusions

A review of studies investigating MT indicates that this rather common phenomenon is reflected in various forms along the mental health continuum, with its persistence across time and culture cementing its role as an inherent part of human experience. In the modern age, TAF specifically may find a more subtle expression in various aspects of cognition, emotion and behaviour, such as misattribution of intention, emotion contagion, and various practices associated with “mind over matter”, from the more mundane (e.g. positive thinking) to the spectacular (e.g. ritualistic magic). Various relationships, such as that between TAF and religious faiths, emphasise the relevance of culture, ethics and spirituality in clinical practice. Important questions that remain to be answered include how MT is related to broader cognition and social skills, as well as the likely influence of developmental factors, including attachment. Aside of psychiatric symptoms, MT can be linked with strengths such as creativity, as well as consideration for others and moral conduct. Future research should explore its utility as a transdiagnostic marker for mental health risk and recovery. Emerging relationships with stress, mood and social connection, hint at the possibility of an adaptive role for MT in terms of psychological coping, which may challenge its assumed function as a straightforward maladaptive marker of psychopathology.

## Data Availability

The original contributions presented in the study are included in the article/**Supplementary Material**. Further inquiries can be directed to the corresponding author.
