# Supplementary material for: The many manifestations of magical thinking: a systematic review
Source: Front Psychiatry. 2026 May 20;17:1759906. doi: 10.3389/fpsyt.2026.1759906 (PMC13230226; doi:10.3389/fpsyt.2026.1759906)
Supplement: Supplementary file 1 [file Table1.docx]

**Supplementary Table 1. Studies of magical thinking organised by secondary topic (n=191)**

| TOPIC | STUDY | STUDY REF | MEASURE | SAMPLE (pps; country) | MAIN FINDINGS |
| --- | --- | --- | --- | --- | --- |
| OCD/traits | Rachman et al (1996) | 206 | TAFS, TAF induction | NU with some evidence of TAF; CA | TAF LO predicted estimates of likelihood, high anxiety and feelings of responsibility, but didn't predict feelings of guilt, morality or urges to neutralise after induction. |
|  | Shafran et al (1996) | 2 | TAFS | NU; CA | TAF was higher in obsessional than in nonobsessional, especially TAF L. TAF-LO was linked to checking. |
|  | Emmelkamp & Aardema (1999) | 51 | TAFS | NC; NL | TAF associated with OCS |
|  | Rassin et al (2000) | 48 | TAFS | NU; NL | TAF triggers thought suppression, which triggers OCD symptoms |
|  | Smari & Holmstein (2001) | 49 | TAFS | NU; IS | TAF found to have a similar role to responsibility and thought suppression in maintaining OCS |
|  | Einstein and Menzies (2004b) | 207 | MIS | NU; AT | MI most strongly related to OCS, can explain links with TAF, superstition |
|  | Einstein and Menzies (2004a) | 3 | TAFS, MIS | OCD; AT | MIS most strongly related to symptoms of OCD patients, other relationships disappeared when MI held constant (TAF, superstition) |
|  | Gwilliam et al (2004) | 6 | TAFS, own Qs | NC; UK | TAF correlated with perceptions of responsibility, as well as OCD symptoms more generally |
|  | Farrell & Barrett (2006) | 208 | TAFS | 6-11; 12-17; NC 18-66, OCD; AT | No difference for children and adults, although the childrens' OCD symptoms were milder and less distressing |
|  | Nelson et al (2006) | 43 | YBOCS, TAFS | OCD; US | TAF-M related to scrupulosity |
|  | Zucker et al (2006) | 56 | TAFS | OCS, young; US | CBT workshop. TAF had reduced at 1 and 5 mth follow up |
|  | Altin & Gencoz (2007) | 46 | TAFS | NU; TR | TAFS correlated with OCD symptoms and thought suppression but not depression. |
|  | Belloch et al (2007) | 50 | TAFS | NC; ES | TAF probability related to frequency of obsessive intrusive thoughts |
|  | Bocci & Gordon (2007) | 209 | MIS, TAFS, TAF induction | NU; UK | Higher neutralising group showed more TAF L, but not higher OCS |
|  | Marcks & Woods (2007) | 210 | TAFS, TAF induction | NU; US | Thought suppression mediated the link between TAF and OCS |
|  | Matthews et al (2007) | 211 | TAFS | NC Adolesc. 13-16; UK | Inflated responsibility completely mediated the effect of TAF on OCS |
|  | Samuels et al (2007) | 66 | CYBOCS | OCD +/- hoarding; US | MT was related to hoarding, miserliness, preoccupation with details, difficulty making decisions, odd behavior or appearance |
|  | Storch et al (2007) | 65 | CYBOCS | Youths; US | Higher MT in hoarders versus nonhoarders |
|  | Verhaak and De Haan (2007) | 29 | C-YBOCS, MTQ | 8-12; 13-18, OCD; US | MT not linked to OCD severity. The sample had low MT scores. |
|  | Einstein and Menzies (2008) | 57 | MIS | OCD; AT | OCD symptoms improvement after CBT (OCI-R/Padua Inv) correlated with change in MT |
|  | Hanstede et al (2008) | 212 | TAFS | NU with OCS; NL | TAF decreased after mindfulness intervention for OCD symptoms |
|  | O'Kearney & Nicholson (2008) | 35 | TAFS | NC; AT | High TAF associated with more emphasis on certainty rather than source of information on lexical task |
|  | Valentiner & Smith (2008) | 25 | TAFS | NU; US | The interaction between shame-proneness, TAF M and obsessions predicts compulsions. The relationship between obsessions and compulsions was stronger as a function of shame-proneness and TAF M |
|  | Storch et al (2009) | 54 | TAFS | OCD; US | TAFS scores rather high but didn't correlate with disability measure |
|  | Twohig et al (2009) | 213 | TAFS | OCD MZ twins (two pairs); US/CA | Mixed evidence of reduction of TAF after intervention |
|  | Calleo et al (2010) | 214 | TAFS, OBQ | OCD; US | TAF predictive of obsessions when worry controlled |
|  | Rees et al (2010) | 4 | TAFS, MIS | NU; AT | MI stronger relationshipe with OCD than TAF |
|  | Twohig et al (2010) | 215 | TAFS | OCD; US | ACT did seem to reduce TAF but progressive relaxation did not |
|  | Yorulmaz et al (2010) | 26 | TAFS, OBQ | NU; TR/CA | Higher TAF M in Turkish sample. OBQ TAF L associated with more OCS in both samples. |
|  | Altin & Gencoz (2011) | 47 | TAFS | NU; TR | TAF-M associated with inflated sense of responsibility, TAF-L associated with thought suppression efforts, and in turn these factors increased OCS |
|  | Besiroglu et al (2011) | 216 | TAFS | OCD; TR | TAFS M was lower after SSRIs only when pps with religious obsessions were included. Changes in TAFS were linked to obsessions rather than compulsions |
|  | Coleman et al (2011) | 53 | TAFS | NU; US | Object checking related to TAF LS versus interpersonal checking more relevant to GAD |
|  | Einstein et al (2011) | 217 | TAFS, MIS | OCD; AT | Treatment trial aimed to reduce MT found decrease in some TAFS scores and e.g. superstition on Padua Inventory |
|  | Jonsson et al (2011) | 58 | TAFS | OCD; DK | Higher TAF-M predicted worse treatment outcome in relation to group CBT specifically |
|  | Yorulmaz et al (2011) | 24 | TAFS | NU; TR | TAF L significantly associated with paranormal beliefs when controlling depression and anxiety |
|  | Yorulmaz & Isik (2011) | 27 | TAFS | NC; TR/BG | Compared to Turkish, lower TAF in remigrants (esp. TAF M), and lower TAF L in Bulgarian sample. TAF M stronger predictor of OCS for Turkish sample but TAF L better predictor for other groups. |
|  | Aydin et al (2012) | 218 | TAFS | NU; TR | SEM showed TAF was one vulnerability agent in the development of OCS |
|  | Farrell et al (2012) | 32 | TAFS | 7-15 OCD, plus mothers; AT | Found maternal metacognition/TAF was positively linked to TAF/thought suppression in younger children. |
|  | Helgadottir et al (2012) | 28 | MIS, TAFS | NC; IS | High MT associated with OCS. Icelandic sample higher MT but lower TAF-M versus Australian sample. |
|  | McNicol & Wells (2012) | 219 | TFI | NU; UK | TAF and thought object fusion predicted symptoms and distress |
|  | Berman et al (2013) | 149 | TAF induction, III | NU; US | One week after TAF induction TAF M and L and urges to neutralise had dissipated |
|  | Bailey et al (2014) | 52 | TAFS | NU; US | TAF self seemed more specific to OCD than anxiety or depressive symptoms |
|  | Fernandez de la Cruz et al (2013) | 30 | C-YBOCS | 7-18, OCD; UK | Higher MT scores in children with sexual obsessions, but higher overall MT. |
|  | Goods et al (2014) | 34 | IBI | NC; INT (mainly AT) | MT partially mediated the relationship between inferential confusion and OCS |
|  | Jones and Bhattacharya (2014) | 71 | MIS, TAF induction | NC, High v low OCD; UK | Precuneus activity associated with self-reported TAF, and higher in high OCD group |
|  | Selles et al (2014) | 31 | CYBOCS | OCD child; US | More MT in the older children (10-18) versus younger (3-9) |
|  | Noorian et al (2015) | 64 | TAFSQ-A, OBQ | NC Adolesc 13-16; ES | TAF likelihood predicts superstition and mental compusions. TAF predicts OCS after controlling for depression |
|  | Ekinci and Ekinci (2016) | 36 | TAFS | OCD, NU; TR | In OCD, TAF-M was positively related to self certainty, but negatively to self-reflectiveness |
|  | Reuman et al (2017) | 37 | TAFS | NU; US | TAF L associated with contamination, unacceptable thoughts, responsibility but not symmetry |
|  | Siwiec et al (2017) | 220 | TAFS | NC with intrusive thoughts; US | Showed cognitive bias modification can reduce TAF |
|  | Fergus & Rowatt (2018) | 40 | TAFS, TAF induction | NU; US | TAF was associated with mental contamination (e.g feeling dirty, need to neutralize) after induction |
|  | Jacoby et al (2018) | 39 | C-TAF | NU; US | C TAF scale correlated with other measures of contamination e.g. contamination cognitions |
|  | Fite et al (2020) | 45 | IBI | NU; US | MT as a mediator of OCD symptoms predicted by greater perceptions of responsibility, threat, or importance of controlling thoughts. |
|  | Kim & Lee (2020) | 62 | TAFS | OCD, NC; KR | DOCS unacceptable thoughts positively predicted by TAF LO but negatively predicted by TAF LS. No such effects for OCI-R. |
|  | Pennequin et al (2020) | 33 | KIDCOPE | 8-12 NC; FR | MT was linked to metacognition and solving problems |
|  | Coughtrey et al (2021) | 41 | TAFS, VOCI-MC | OCD; UK | Mental contamination was common and related to specific TAF and more general TA |
|  | Lee et al (2021) | 69 | TAFS, TAF induction | NC; KR | Negative TAF, relative to positive TAF, recruits insula, med PFC, precuneus, GP and thalamus |
|  | Marazziti et al (2021) | 221 | MT own questions | NC (tennis, non-tennis); IT | More MT in athletes. 23/25 exhibited MT and superstitious behaviours e.g. colour of clothes |
|  | Benatti et al (2022) | 55 | YBOCS | OCD; IT | MT remained in the milder state i.e. for patients who were considered to have improved over time |
|  | Cares et al (2022) | 222 | TAFS | NU; US | TAF induction task linked to anxiety in general rather than just TAF |
|  | Fite & Magee (2022) | 223 | IBI, TAF (IT) induction | NU (33% over OCI-R cutoff); US | MT related to worry after induction rather than neutralisation, responsibility etc. |
|  | Jellinek et al (2022) | 224 | TAF induction, TAF Qs | NC Therapists; DE | TAF (more avoidance and neutralisation after induction) was related to therapists less likely to use ERP as OCD therapy |
|  | Lee et al (2022) | 70 | TAFS, TAF induction | OCD, NC; KR | Higher TAF in OCD; salience network showed lower power in low freq domain of salience network during TAF induction |
|  | Lee et al (2022) | 72 | TAFS, OBQ, TAF induction | OCD, NC; KR | MCC -left insula FC positively correlated with TAF in the OCD group, as well as responsibility |
|  | Burhan et al (2023) | 59 | TAFS | OCD; TR | Lower TAF one of the predictors of effectiveness of CBGT for OCD |
|  | Hansmeier et al (2023) | 42 | TAFS, TAF induction | NC, inc. OCD; DE | Induction increased TAF and shame, related to attitude to violence as induction was violence, but not related to TAF trait beliefs or religiosity |
|  | Lee et al (2023) | 44 | TAF induction | OCD, NC; KR | TAF scores higher for OCD group. Correlation between TAF and RT in HC but not OCD. OCD RT correlated with guilt. |
|  | Ouellet-Courtois & Radomsky (2023) | 38 | TAFS | NU; CA | Moral disgust stimuli elicited greater TAF than core disgust especially in those high in contamination fear |
|  | Cetin et al (2024) | 67 | TAFS, MIS | OCD v HC; TR | Higher TAF in OCD group. High MIS scores linked to obsessions and hoarding |
|  | Farouk et al (2024) | 68 | SPQ | OCD; EG | MT correlated with OCD symptoms related to aggression and global distress |
|  | Lee et al (2024) | 73 | TAFS, TAF induction | OCD; KR | Precuneus connectivity occurred alongside changes in TAF scores |
| SSDs/traits | Raine (1992) | 23 | SPQ | NU; US | Females higher OBMT than males |
|  | Poreh et al (1993) | 92 | MIS | NU; US | The schizotypy scales correlated with high scores on the nonverbal portion of the creativity tests and with a left ear preference to dichotically presented verbal stimuli |
|  | Roth and Baribeau (1997) | 77 | SPQ | NU; CA | More MT in females versus males |
|  | Sobin et al (2000) | 97 | SIS, mod. | OCD; US | MT absent in 39% of sample and otherwise usually mild-moderate |
|  | Chang and Lenzenweger (2001) | 81 | SPQ | SZ FDR, NC; US | MT/OB scores associated with poor performance on 2 point somatosensory distinction task |
|  | Torgersen et al (2002) | 111 | SIB | SZ, FDR (inc. twins), NC, Axis I/II disorder; NO | MT high among the MZ twins of those with SPD |
|  | Dickey et al (2003) | 115 | SCID II | NC (inc SPD); US | MT specifically associated with smaller right fusiform volumes in SPD |
|  | Mason et al (2004) | 102 | SPQ | HR PSYCH; AT | MT items some of the most predictive of transition to psychosis |
|  | Stefanis et al (2004) | 79 | SPQ | Young army conscripts; GR | Higher MT in young men from urban v rural areas |
|  | Lee et al (2005) | 100 | TAFS | NU; US | The relationship between TAF L and OCD symptoms was significantly attenuated after controlling for schizotypal traits. TAF M demonstrated negligible association with OCD, depression, or schizotypal traits. |
|  | Berle et al (2006) | 201 | TAFS, MIS | SZ, NC; AT | Higher ratings for TAF L, magical ideation and OC symptoms in SZ |
|  | Yung et al (2006) | 110 | CAPE | Youth psychiatric service, non-psychotic; AT | MT not associated with poor functioning unless with distress |
|  | Kabakci et al (2008) | 101 | TAFS, MIS | SZ, NC; TR | Patients higher scores on TAF L Others but not on Self; tendency to get higher scores on TAF M |
|  | Corlett et al (2009) | 89 | MIS | NC; UK | High MT linked to false memories on word list task |
|  | Yung et al (2009) | 109 | CAPE | NU; AT | MT only weakly related to functioning and depression |
|  | Steel et al (2009) | 85 | STA | NU; UK | Experience of PA/SA not linked to high MT (but was to paranoia and UPE) |
|  | Muris and Merckelbach (2010) | 90 | TAFS | NU; NL | Positive correlations between TAF and various aspects of schizotypy but not after controlling for fantasy proneness |
|  | Fonseca-Pedrero et al (2011) | 99 | ESQUIZO-Q | NC adolesc.; ES | Prevalence of 43% of MT in the community sample |
|  | Armando et al (2012) | 98 | CAPE | NU; IT | MT was identified in 64.3% of the sample. |
|  | Fonseca-Pedrero et al (2012) | 74 | ESQUIZO-Q | NC; ES | Greater MT for females versus males |
|  | Ribolsi et al (2013) | 95 | SPQ | NC; IT | High MT associated with reduced leftward bias on line bisection. |
|  | Bedwell et al (2013) | 94 | SPQ | BIP, SSD; US | VEP study -reduced P1 amplitude on low contrast/neutral condition associated with high MT |
|  | Collip et al (2013) | 84 | CAPE | NC, adolesc.; AT | MT less predictive of interpersonal functioning than other measured variables e.g.bizarre experinece; persecutory ideation |
|  | Barron et al (2014) | 103 | SPQ | NC; INT (mainly US, UK) | MT predicted conspiricist ideation |
|  | Garcia-Montes et al (2014) | 8 | MIS | SZ, OCD, NC; ES | MI scores differentiated patients with SZ with and without auditory hallucinations |
|  | Dasse et al (2015) | 88 | MIS | NU; US | Higher scores on the MIS predicted higher hyponotyzability. Also associated with dissociative experience, creative experiences |
|  | Wiltink et al (2015) | 225 | CAPE | NU; AT | High MT associated with Extraversion, Openness to Experience, Agreeableness, and Conscientiousness (usually positively) |
|  | Mededovic and Dordevic (2017) | 91 | DELTA-10 | NU (Painters v non-painters); RS | MT more pronounced in painters |
|  | Barron et al (2018) | 105 | SPQ | NC; INT (mainly US, UK, IN) | Cognitive insight mediated the link between MT and IOR. MT was also associated with need for cognition. |
|  | Bedwell et al (2018) | 96 | SPQ | BIP, SSD; US | VEP reduced P1 amplitude on low contrast condition in association with MT across the clinical samples |
|  | Mimarakis (2018) | 78 | STA | NC sec sch; GR | Urbanicity predicted MT in females |
|  | Weintraub et al (2018) | 106 | SPQ | NC young adults; US | Hoarding linked to MT |
|  | March and Springer (2019) | 104 | SPQ | NU; AT | Magical thinking predicted beliefs in conspiracy theory |
|  | Turley et al (2019) | 108 | CAPE | NU; AT | Positive significant correlation between perceived stress and MT but much less strong than e.g. persecutory ideation |
|  | Escola-Gascon et al (2020) | 87 | CAPE | NC; ES | Suggests social quarantine increased certain SZ traits inc MT |
|  | Khaled et al (2020) | 75 | SPQ | NC (arab v non-arab); QA | Arab ethnicity, female gender, and psychological distress were linked to psychotic experiences through associations with OBMT |
|  | Dalal et al (2021) | 82 | SPQ | NU; CA | MT linked to multisensory temporal processing |
|  | Eddy & Hansen (2021) | 83 | TAFS | NU; UK | TAF M was predicted by emotion contagion, alexithymia and need for closure. TAF L was predicted by personal distress, sense of agency and alexithymia. |
|  | Elek et al (2021) | 112 | SPQ | NU; HU | The AA variant of the SNP rs6913660 on the HIST1H2BJ gene was associated with higher intensity of magical thinking. |
|  | McDonald et al (2021) | 93 | SPQ | NC; UK | MT significantly positively correlated with positive ‘flow’-type experience of creativity, namely Distinct Experience, Absorption, and Power/Pleasure |
|  | Chau et al (2022) | 86 | SPQ | NC (young); CN | After controlling for depression and anxiety, MT negatively associated with loneliness |
|  | Saarinen et al (2022) | 113 | TCI (ST) | HR SZ, LR SZ (NC); FI | Polygenic risk score high MT in those at high risk over time but MT falls in the low risk group |
|  | Speck and Witthoft (2022) | 80 | SPQ | IEI (idiopathic enviroment intolerance); DE | High MT linked to modern health worries (e.g. radiation from phones) |
|  | Wastler and Lenzenweger (2023) | 107 | MIS | NU; US | No link between MT and suicide risk |
|  | Garner et al (2024) | 114 | SPQ | NC (Ketogenic diet v other diet); US | Lower incidence of MT for those on ketogenic diet |
| Anxiety and mood disorders | Coles et al (2001) | 116 | TAFS | NU; US | TAFS not associated with worry in general, moreso with OC traits |
|  | Rassin et al (2001) | 128 | TAFS | OCD, ANX; NL | No association between TAF and thought suppression. NSD in TAF between groups. TAF scores did not predict treatment response. |
|  | Abramowitz et al (2003) | 200 | TAFS | OCD, ANX, NC; US | OCD higher on LS and LO but linked to negative affect, which mediates the relationship between L TAF and OCD. L TAF linked to anxiety but moral TAF linked to depression. |
|  | Barrett & Healey (2003) | 226 | C-YBOCS, Idiographic assessment task | NC, OCD, ANX, child; AT | Significantly higher ratings of TAF in OCD than HC but not compared to anxiety |
|  | Presson & Benassi (2003) | 117 | MIS | NU; US | MT positively associated with depressive signs |
|  | Libby et al (2004) | 227 | TAFS | NU young, OCD, ANX; UK | Higher TAFS in OCD v other groups. |
|  | Einstein and Menzies (2006) | 228 | MIS | OCD, PAN; AT | No diff for MI. Higher MT in cleaners vesus checkers. |
|  | Yorulmaz et al (2008) | 63 | TAFS | NC, OCD, ANX; TR | TAFS M and L associated with OCS in Turkish sample. Higher TAF L in OCD v controls, but no group differences for TAF M |
|  | O'Leary et al (2009) | 229 | TAFS, YBOCS | OCD, ANX, HC; NZ | TAF and OCS were only correlated if high negative affect |
|  | Belloch et al (2010) | 118 | OBI(S) | NC, AD, MDD; ES | OCD higher scores for TAF items than anxiety disorders group but not the depessive group. |
|  | Hausteiner-Wiehle and Sokollu (2011) | 129 | SPQ | C-DER, SOMAT; DE | Higher MT in somatoform group e.g. more likely to believe in telepathy or clairvoyance |
|  | West & Willner (2011) | 126 | MIS | NC, OCD, GAD; UK | OCD and GAD linked to TAF. GAD duration positively associated with MT. |
|  | Brown & Naragon-Gainey (2013) | 123 | TAFS | C-ANX; US | TAF scores only associated with OC traits in the sample, not anxiety or mood symptoms |
|  | Gahmari-Kivi et al (2013) | 120 | TAFS | OCD, DEP; IR | High TAF-M in MDD as well as in OCD |
|  | Thompson-Hollands et al (2013) | 125 | TAFS | C-ANX, inc. OCD, ANX; US | The presence of any GAD diagnosis was the strongest predictor of likelihood TAF |
|  | Gjelsvik et al (2018) | 122 | TAFS modified for suicidal depression | NC, DEP, DEP+SUI; UK | Mood induction increased TAF in DEP, who showed higher TAF overall and negative uncontrollable TAF, but lower positive controllable TAF v NC. |
|  | Arnaez et al (2021) | 127 | IITI | IAD, NC; ES | In illness anxiety disorder, overestimation of threat and TAF contributed to intrusive thoughts. |
|  | Olivares-Olivares et al (2022) | 230 | OBI(S)r | OCD, ANX, TTM, NC; ES | All clinical groups higher TAF L than controls. OCD and TTM higher TAF M than HC, TTM also higher TAF M than anx dis and OCD when mood was controlled. |
|  | Lee et al (2024) | 121 | TAFS, TAF induction | DEP, NC; KR | Higher TAF-L in MDD. More activity in MDD for TAF in areas such as caudate, IFG/insula, putamen and IPL, which they suggest is linked to high saliency and habit formation. |
|  | Shams et al (2024) | 124 | IITIS | DEP, OCD, NC; IR | All had unwanted intrusive thoughts but only OCD high TAF |
|  | Lee et al (2025) | 119 | TAFS | OCD, DEP, HC; KR | Higher TAFL in OCD and MDD v HC. No diff for OCD and MDD. |
| Eating Disorders | Radomsky et al (2002) | 130 | TAFS, TSF induction | ED; UK | Neutralizers of TSFI showed higher TAFS scores. |
|  | Roncero et al (2011) | 131 | TAFS | ED-OCD, NC; ES | BN purgative patients showed higher TAF-L than healthy controls; and binge eating patients showed lower TAF-M |
|  | Garcia-Soriano et al (2014) | 133 | OITI, TAFS | ED, OCD; ES | TAF apparent in both groups, and predicted emotional disturbances. |
|  | Lee et al (2020) | 132 | TAFS | ED; US | All types of TAF related to ED severity and tracked recovery. |
|  | Pullmer et al (2020) | 134 | TAFS | NC adolesc; CA | TAF related to depression, but didn’t explain a significant proportion of it in comparison to thought shape fusion, eating pathology and OCS |
| Gambling compulsions | Teed (2012) | 137 | GBQ | NU; CA | Beliefs in luck transferrence associated with problematic gambling |
|  | Savage (2014) | 136 | MIS | NC, twins; AU | MT related to more problematic real life gambling behaviours |
|  | Passanisi (2017) | 135 | MIS | NC, adolesc; IT | MT related to risky/illogical gambling decisions |
| PDs: Borderline personality disorder | Zanarini et al (2013) | 138 | Interview for borderlines (covers "odd thinking" | BPD, CC = PDs; US | More evidence of MT in borderline personality compared to other PDs (Axis II) |
|  | Alesiani et al (2014) | 140 | TCI (ST) | BPD, BPD traits + other PD and DEP; IT | High MT predicted increased likelihood of CBT dropout |
| Spirituality or religion | Rassin and Koster (2003) | 142 | TAFS | NU (CHR-P, CATH); NL, BE | Protestants reported more religious than catholics and higher TAF-M. TAF-L negatively correlated with religiosity in the protestant group. |
|  | Yorulmaz et al (2009) | 147 | TAFS | CHR, MUS; TR | For Christians, more religious were higher TAF-M. TAF M was associated with religiosity in both groups. |
|  | Berman et al (2010) | 149 | TAFS, TAF induction | CHR-P, AGN/ATH; US | Christians higher TAF L and more neutralisation for task, but only specifically higher TAF M on the TAFS |
|  | Siev et al (2010) | 145 | TAFS | NC (CHR, JEW); US | Christians endorsed higher levels of TAF M than did Jews; religiosity was correlated with TAF M in Christians only; and TAF M was related to OCD symptoms only in Jews |
|  | Unterrainer et al (2011) | 152 | MIS | NU; AU | MT related to connectedness in a study of religious and spiritual wellbeing |
|  | Berman et al (2013) | 149 | TAFS | NU; US | Religion-related variables predicted TAF M whereas parenting strategies were associated with TAF L |
|  | Cougle et al (2013) | 150 | TAFS, own intentionality and moral TAF measure | NU; US | Perception of the immorality of intentional negative thoughts was associated with Protestant/Catholic affiliation and greater prayer frequency. Neither form of Moral TAF was associated with OCD symptoms. |
|  | Deacon et al (2013) | 144 | TAFS | Clergy; US | Pastors affiliated with the more conservative denomination evidenced higher moral TAF, and this in turn fully mediated responses to a scenario about a scrupulous parishioner aligned with compulsive rituals (e.g. confession) |
|  | Williams et al (2013) | 151 | TAFS, TAF induction | CHR, JEW, AGN/ATH; AT | Christianity moderated the effects of religiosity on moral TAF beliefs, which in turn mediated the relationship between religiosity and obsessive-compulsive symptoms. |
|  | Fergus et al (2014) | 143 | TAFS | CHR-P, CATH; US | NSD between groups but TAF was positively associated with religiousity and negative affect |
|  | Inozu et al (2014) | 159 | TAFS | NC (MUS); TR | The relationship between religiosity and OC symptoms was mediated by TAF and disgust sensitivity. |
|  | Breslin & Lewis (2015) | 156 | STA | NC; IE | MT was positively correlated with prayer, especially ritual and meditative prayer. |
|  | Eremsoy & Inozu (2016) | 162 | MIS | NC; TR | TAF scores correlated with scores on OCI-R. MT mediated the relationship between OCS and religiosity. |
|  | Mauzay et al (2016) | 160 | TAFS | NU; US | TAF M mediated associations between religiosity & OCD. TAF L mediated the relationship between paranormal beliefs & OCD. |
|  | Breslin & Lewis (2017) | 157 | STA | NC; IE | MT predicted measures of mysticism and prayer (e.g. experience of God) |
|  | Siev et al (2017) | 146 | TAFS | NC (MUS, JEW); IL | Moral TAF was higher in muslims and related to scrupulosity across the entire sample. |
|  | Jones et al (2019) | 231 | TAFS | Imams; IR, AT | Sunni imams had significantly higher scores than Shia imams on TAF subscales. |
|  | Kiken (2019) | 158 | IBI | NC; US | TAF was uniquely associated with mind-body therapy use. |
|  | Younce and Wu (2020) | 161 | TAFS, TAF induction | NU (CP); US | TAF L associated with anxiety and urge to neutralise but TAF M only with perceived moral wrongness |
|  | Fite et al (2021) | 155 | TAFS | NC (LIB, CONS); INT | Moral vitalism was found to mediate therelationship between religiosity and TAF-M in a sample comprised of mostly political liberals and conservatives. |
|  | Henderson et al (2022) | 154 | TAFS | OCD, NC; CA | Religious crisis was positively associated with TAF in both groups |
|  | Kumar et al (2025) | 153 | SPQ | NU; IN | MT influenced variables linked to life satisfaction such as religious belonging and intolerance of uncertainty. |
|  | Namdiero-Walsh et al (2022) | 148 | Q specifically about MT practices e.g. rituals | NC; TR, KR, IN, DE | Religiosity, along with country and gender influenced motives and reactions to MT related practices |
| Cognitive factors | Keinan (1994) | 165 | MT own questions | NC; IL | MT more common in participants who experienced high-stress condition, especially if low tolerance of ambiguity as trait. |
|  | Zucker et al (2002) | 170 | TAF questions, Intrusive thought induction | NC (high TAF); US | Psychoeducation reduced anxiety, TAF scores and urge to neutralise after thought induction. |
|  | Beitel et al (2004) | 164 | MIS | NU; US | Psychological mindedness (using thoughts, feelings and behaviours to try to understand the self and others) was inversely related to MT. |
|  | Marino et al (2008) | 163 | ROII, TAFS, MIS | NU; US | Religiosity, as predicted by ethnic identity (in turn mediated by inflated sense of responsibility) predicted TAF. |
|  | Marino Carper et al (2010) | 169 | TAFS | NU (with high TAF); US | Psychoeducation reduced TAF scores, maintained at 2 weeks. |
|  | Ching and Tang (2016) | 171 | TAFS, cognitive dissonance protocol | NC; SG | Adding mock EEG-based computer trials that induced cognitive dissonance about TAF beliefs, prior to TAF-specific psychoeducation, enhanced and maintain the reduction of TAF beliefs. |
|  | Zhu et al (2017) | 168 | TAFS | NC; CN | TAF-L (but not TAF-M) mediated the relation between inflated responsibility and OC symptoms. |
|  | Jimenez-Ros et al (2020) | 172 | OBI, TAF induction | NC; PT | Reading about thought control reduced TAF-M versus reading a neutral abstract, but thought intrusiveness was not affected |
|  | Siev et al (2022) | 167 | TAFS, TAF-M induction | NU; US | Inflated responsibility beliefs predicted distress and moral emotions. Beliefs about the importance of thoughts predicted moral emotions and neutralizing. |
|  | Rezaei et al (2023) | 166 | TAFS | NC; IR | High prevalence of relationship related OCD linked to TAF, OCD severity and attachment anxiety and avoidance. |
| Early trauma | Selvi et al (2012) | 177 | TAFS | OCD; TR | No relationship found between TAF and childhood trauma Q. |
|  | Berman et al (2013) | 173 | TAFS, TAF induction | NU; US | Emotional abuse and physical neglect predicted TAF-L, whereas physical abuse predicted TAF-M |
|  | Velikonja et al (2019) | 176 | SPQ | SPD, NC; US | Childhood trauma associated with SPD generally, cognitive perceptual factors inc. MT most strongly associated with sexual abuse |
|  | Lu et al (2020) | 175 | CAPE | NU; CN | Positive correlations between MT and all three kinds of abuse (P, E, S) and physical neglect, but a negative correlation between MT and emotional neglect. |
|  | Lee et al (2024) | 174 | TAFS, TAF induction during fMRI | NC (young); KR | Exaggerated TAF in CM group linked to increased response of AI and dACC. Emotional abuse related to higher TAF L. |
| Neurophysiological studies | Bell et al (2007) | 19 | Apophenia measure, MIS | NC; UK | TMS over left lateral temporal area reduced tendency to report seeing meaning in "random noise" type images |
|  | Brugger et al (2007) | 179 | MIS | NC (split into high and low MT); CH | Suggest that greater MT associated with reduced LH dominance (reduced right side memory/attention) |
|  | Lee et al (2019) | 178 | TAF induction sentences, TAFS, MRI | NU; KR | MRI showed precuneus activation in association with TAF. |
|  | Narmashiri et al (2022) | 180 | PBSr, EEG | NU; IR | PBS (in MT in terms of superstitions, precognition) related to less front activity and various patterns of coherence |
| Developmental | Bolton et al (2002) | 181 | MT own questions | 5-17 NC child/adolesc; UK | Association with anxiety/OCD but stronger in boys than girls. MT reduced around age 12 but then increased again. |
|  | Evans et al (2011) | 182 | Own scale: TAF instrument for children | 7-14 NC child/adolesc; US | TAF predicted compulsive like behaviour in older children. MT seemed to reduce with age. |

**KEY**

ADOL: adolescents; AGN: agnostics; ANX; Anxiety disorder; AT: Australia; ATH: atheists; AU: Austria; BE: Belgium; BG: Bulgaria; CA: Canada; CAPE: Community assessment of psychic experiences (242); C-ANX: Clinical sample, anxiety disorders; CATH: Catholics; C-DER: Clinical sample, dermatology; CH: Switzerland; CHR: Christians; CN: China; CON: Conservatives; C-TAF: contamination TAF scale (237); C-YBOCS: Child version of YBOCS (233); DE: Germany; DELTA-10: Inventory for the assessment of dysregulation (245); DEP: depressive disorder; DK: Denmark; DOCS: Dimensional obsessive-compulsive scale (61); EG: Egypt; ESQUIZO-Q: Oviedo Questionnaire for Schizotypy Assessment (244); FI: Finland; FR: France; GAD: Generalized anxiety disorder; GBQ: Gamblers beliefs questionnaire (249); GR: Greece; IAD: Illness anxiety disorder; IBI: Illusory Beliefs Inventory (236); IE: Ireland; III: Interpretation of intrusions inventory (234); IITI: Ilness intrusive thoughts inventory (246); IITIS: International intrusive thoughts schedule (247); IL: Israel; IN: India; INT: international study; IR: Iran; IS: Iceland; KIDCOPE: Childrens Coping Behavior Questionnaire (238); KR: LIB: Liberal; South Korea; MIS: Magical Ideation Scale (5); MT: Magical thinking; MTQ: Magical thinking questionnaire (181); MUS: Muslims; NC; non-clinical/community source; NL: Netherlands; NO: Norway; NU; non-clinical university source; NZ: New Zealand; OBI(S): Obsessive beliefs inventory, Spanish translation (118); OBQ: Obsessive beliefs questionnaire (234); OCD: Obsessive compulsive disorder; OCI-R: Obsessive Compulsive Inventory-revised (60); OCS: milder OCD symptoms in undiagnosed; OITI: Obsessive intrusive thoughts inventory (248); PAN: Panic disorder; PBSr: Paranormal Belief Scale revised (251); PD: Personality disorder; PT: Portugal; QA: Qatar; ROII: Revised obsessive intrusions inventory (250); RS: Serbia; SCID II: Structured clinical interview for DSM-IV (241); SG: Singapore; SIB: Schedule for interviewing borderlines (240); SIS: Structured interview for schizotypy (239); SPQ: Schizotypal Personality Questionnaire (23); SSD: Schizophrenia spectrum disorders; STA: Schizotypy personality scale (243); SZ: Schizophrenia; TAF; Thought action fusion; TAF-L: TAFS likelihood subscale; TAF-M: TAFS moral subscale; TAFS: Thought Action Fusion Scale (2); TAFQ-A: Thought action fusion questionnaire for adults (64); TCI: Temperament and character inventory (139); TFI: Thought fusion instrument (235); TR: Turkey; UK: United Kingdom; US: United States of America; VOCI-MC: Vancouver OCI – mental contamination scale (252); YBOCS: Yale-Brown Obsessive Compulsive Scale (232).

*Note: Scale references provided in brackets.* *Country acronyms are as per ISO 3166-1 alpha-2 codes. Sample age range is adults unless specified.*

*ADDITIONAL REFERENCES (TO THOSE LISTED WITHIN THE PAPER)*

206. Rachman S, Shafran R, Mitchell D, Trant J, Teachman B. How to remain neutral: An experimental analysis of neutralization. BEHAVIOUR RESEARCH AND THERAPY. 1996;34(11–12):889–98.

207. Einstein D, Menzies R. Role of magical thinking in obsessive-compulsive symptoms in an undergraduate sample. DEPRESSION AND ANXIETY. 2004a;19(3):174–9.

208. Farrell L, Barrett P. Obsessive-compulsive disorder across developmental trajectory: Cognitive processing of threat in children, adolescents and adults. CRITISH JOURNAL OF PSYCHOLOGY. 2006;97:95–114.

209. Bocci L, Gordon P. Does magical thinking produce neutralising behaviour? An experimental investigation. BEHAVIOUR RESEARCH AND THERAPY. 2007;45(8):1823–33.

210. Marcks B, Woods D. Role of thought-related beliefs and coping strategies in the escalation of intrusive thoughts: An analog to obsessive-compulsive disorder. BEHAVIOUR RESEARCH AND THERAPY. 2007;45(11):2640–51.

211. Matthews L, Reynolds S, Derisley J. Examining cognitive models of obsessive compulsive disorder in adolescents. BEHAVIOURAL AND COGNITIVE PSYCHOTHERAPY. 2007;35(2):149–63.

212. Hanstede M, Gidron Y, Nyklícek I. The Effects of a Mindfulness Intervention on Obsessive-Compulsive Symptoms in a Non-Clinical Student Population. JOURNAL OF NERVOUS AND MENTAL DISEASE. 2008;196(10):776–79.

213. Twohig M, Whittal M, Peterson K. Treatment of Monozygotic Twins with Obsessive Compulsive Disorder Using Cognitive Therapy and Exposure with Ritual Prevention. BEHAVIOURAL AND COGNITIVE PSYCHOTHERAPY. 2009;37(4):475–80.

214. Calleo J, Hart J, Björgvinsson T, Stanley M. Obsessions and worry beliefs in an inpatient OCD population. JOURNAL OF ANXIETY DISORDERS. 2010;24(8):903–8

215. Twohig M, Hayes S, Plumb J, Pruitt L, Collins A, Hazlett-Stevens H, et al. A Randomized Clinical Trial of Acceptance and Commitment Therapy Versus Progressive Relaxation Training for Obsessive-Compulsive Disorder. JOURNAL OF CONSULTING AND CLINICAL PSYCHOLOGY. 2010;78(5):705–16.

216. Besiroglu L, Çetinkaya N, Selvi Y, Atli A. Effects of selective serotonin reuptake inhibitors on thought-action fusion, metacognitions, and thought suppression in obsessive-compulsive disorder. COMPREHENSIVE PSYCHIATRY. 2011;52(5):556–61.

217. Einstein D, Menzies R, St Clare T, Drobny J, Helgadottir F. The treatment of magical ideation in two individuals with obsessive compulsive disorder. COGNITIVE BEHAVIOUR THERAPIST. 2011;4(1):16–29.

218. Aydin A, Boysan M, Tutarel-Kislak S, Kalafat T, Selvi Y, Besiroglu L. The Predictive Value of Interpersonal Schemas, Perfectionism, and Thought Action-Fusion in Obsessive Compulsive Disorder. DUSUNEN ADAM JOURNAL OF PSYCHIATRY AND NEUROLOGICAL SCIENCES. 2012;25(2):108–18

219. McNicol K, Wells A. Metacognition and Obsessive-Compulsive Symptoms: The Contribution of Thought-Fusion Beliefs and Beliefs about Rituals. INTERNATIONAL JOURNAL OF COGNITIVE THERAPY. 2012;5(3):330–40.

220. Siwiec S, Davine T, Kresser R, Rohde M, Lee H. Modifying thought-action fusion via a single-session computerized interpretation training. JOURNAL OF OBSESSIVE-COMPULSIVE AND RELATED DISORDERS. 2017;12:15–22.

221. Marazziti D, Parra E, Amadori S, Arone A, Palermo S, Massa L, et al. Obsessive-compulsive and depressive symptoms in professional tennis players. 2021;18(6):304–11.

222. Cares S, Mangen K, Wu K. An examination of the sentence task through in-person and online administrations. JOURNAL OF OBSESSIVE-COMPULSIVE AND RELATED DISORDERS. 2022;32:1-9.

223. Fite R, Magee J. The role of magical thinking, sensitivity, and thought content in thought-action fusion. JOURNAL OF SOCIAL AND CLINICAL PSYCHOLOGY. 2022;41(2):128–54.

224. Jelinek L, Balzar A, Moritz S, Reininger K, Miegel F. Therapists’ Thought-Action Fusion Beliefs Predict Utilization of Exposure in Obsessive-Compulsive Disorder. BEHAVIOR THERAPY. 2022;53(1):23–33.

225. Wiltink S, Nelson B, Velthorst E, Wigman J, Lin A, Baksheev G, et al. The relationship between personality traits and psychotic like experiences in a large non-clinical adolescent sample. PERSONALITY AND INDIVIDUAL DIFFERENCES. 2015 Jan;73:92–7.

226. Barrett P, Healy L. An examination of the cognitive processes involved in childhood obsessive-compulsive disorder. BEHAVIOUR RESEARCH AND THERAPY. 2003;41(3):285–99.

227. Libby S, Reynolds S, Derisley J, Clark S. Cognitive appraisals in young people with obsessive-compulsive disorder. JOURNAL OF CHILD PSYCHOLOGY AND PSYCHIATRY. 2004;45(6):1076–84.

228. Einstein D, Menzies R. Magical thinking in obsessive-compulsive disorder, panic disorder and the general community. BEHAVIOURAL AND COGNITIVE PSYCHOTHERAPY. 2006;34(3):351–7.

229. O’Leary E, Rucklidge J, Blampied N. Thought-action fusion and inflated responsibility beliefs in obsessive-compulsive disorder. CLINICAL PSYCHOLOGIST. 2009;13(3):94–101.

230. Olivares-Olivares P, Rosa-Alcazar A, Martinez-Esparza I, Rosa-Alcazar A. Obsessive beliefs and uncertainty in obsessive compulsive and related patients. INTERNATIONAL JOURNAL OF CLINICAL AND HEALTH PSYCHOLOGY. 2022;22(3)100316.

231. Jones M, Harris L, Esfahani R. Imams’ Experience With and Response to Mosque-Goers With OCD Scrupulosity. BEHAVIOUR CHANGE. 2019;36(1):29–40.

232. Goodman WK, Price LH, Rasmussen SA, Mazure C, Fleischmann RL, Hill CL, et al. The Yale-Brown obsessive compulsive scale: I. Development, use, and reliability. ARCHIVES OF GENERAL PSYCHIATRY. 1989;46(11):1006–11.

233. Goodman WK, Price LH, Rasmussen SA, Riddle MA, Rappoport JL. Children’s Yale-Brown Obsessive- Compulsive Scale (CY-BOCS). New Haven, CT: Clinical Neuroscience Unit: 1991.

234. Obsessive Compulsive Cognitions Working Group. Development and initial validation of the obsessive beliefs questionnaire and the interpretation of intrusions inventory. BEHAVIOUR RESEARCH AND THERAPY. 2001;39(8):987-1006.

235. Wells A, Gwilliam P, Cartwright-Hatton S. The Thought Fusion Instrument (TFI). Manchester, UK; University of Manchester: 2001.

236. Kingdon BL, Egan SJ, Rees CS. The Illusory Beliefs Inventory: a new measure of magical thinking and its relationship with obsessive compulsive disorder. BEHAVIOURAL AND COGNITIVE PSYCHOTHERAPY. 2012;40(1):39-53.

237. Rachman, SJ. Fear of contamination: Assessment and treatment. Oxford: Oxford University Press: 2006.

238. Spirito A, Stark LJ, Williams C. Development of a brief coping checklist for use with pediatric populations. JOURNAL OF PEDIATRIC PSYCHOLOGY. 1988;13(4):555-74.

239. Kendler KS, Lieberman JA, Walsh D. The Structured Interview for Schizotypy (SIS): a preliminary report. SCHIZOPHRENIA BULLETIN. 1989;15(4):559-71.

240. Baron M. The schedule for interviewing borderlines (SIB). New York State Psychiatric Institute, New York: 1980.

241. First MB, Gibbon M. The structured clinical interview for DSM-IV axis I disorders (SCID-I) and the structured clinical interview for DSM-IV axis II disorders (SCID-II). In Hilsenroth M, Segalaniel DL (Eds.). COMPREHENSIVE HANDBOOK OF PSYCHOLOGICAL ASSESSMENT. 2004. Vol 2. Personality assessment, pp. 134–143. Hoboken, NJ; Wiley.

242. Stefanis NC, Hanssen M, Smirnis NK, Avramopoulos DA, Evdokimidis IK, Stefanis CN, et al. Evidence that three dimensions of psychosis have a distribution in the general population. PSYCHOLOGICAL MEDICINE 2002;32(2):347-58.

243. Claridge G, Broks P. Schizotypy and hemisphere function: I. Theoretical considerations and the measurement of schizotypy. PERSONALITY AND INDIVIDUAL DIFFERENCES. 1984;5(6):633–648.

244. Fonseca-Pedrero E, Muñiz J, Lemos-Giráldez S, Paino M, Villazón-García U. ESQUIZO-Q: Cuestionario Oviedo para la Evaluación de la Esquizotipia [ESQUIZO-Q: Oviedo Questionnaire for Schizotypy Assessment ]. Madrid: TEA ediciones SA: 2010.

245. Knezevic G, Savic D, Kutlesic V, Opacic G. Disintegration: A reconceptualization of psychosis proneness as a personality trait separate from the Big Five. JOURNAL OF RESEARCH IN PERSONALITY. 2017;70:187–201.

246. Arnaez S, Garcıa-Soriano G, Belloch, A. Hypochondriasis and illness-related intrusive thoughts: Development and validation of an evaluation instrument. BEHAVIORAL PSYCHOLOGY, 2017;25 (1):165.

247. RCIF. The International Intrusive Thoughts Schedule, Version 6. Barecelona, Spain: 2007.

248. Garcia-Soriano, G. Obsessional Intrusive Thoughts Inventory (INPIOS). American Psychiatric Association; psychTESTS: 2008.

249. Steenbergh TA, Meyers AW, May RK, Whelan JP. Development and validation of the Gamblers' Beliefs Questionnaire. PSYCHOLOGY OF ADDICTIVE BEHAVIORS. 2002;16(2):143-9.

250. Purdon C, Clark DA. Obsessive intrusive thoughts in nonclinical subjects. Part I. Content and relation with depressive, anxious and obsessional symptoms. BEHAVIOUR RESEARCH AND THERAPY. 1993;31(8):713-20.

251. Tobacyk JJ. A revised paranormal belief scale. INTERNATIONAL JOURNAL OF TRANSPERSONAL STUDIES. 2004;23(1):94-8.

252. Thordarson DS, Radomsky AS, Rachman S, Shafran R, Sawchuk CN, Hakstian AR. The Vancouver Obsessional Compulsive Inventory (VOCI). BEHAVIOUR RESEARCH AND THERAPY, 2004;42(11):1289–1314.
