# Supplementary material for: The many manifestations of magical thinking: a systematic review
Source: Front Psychiatry. 2026 May 20;17:1759906. doi: 10.3389/fpsyt.2026.1759906 (PMC13230226; doi:10.3389/fpsyt.2026.1759906)
Supplement: Supplementary file 2 [file Table2.docx]

**Supplementary Table 2. Quality appraisal of clinical studies on the topic of MT**

| Topic | Study | N sample; sub-samples | Age: Mean (SD), Range | % Female | Ethnicity data | Other sample data | MH scales or screen | MT data presented | MT scores detail (Inc. TAFS specific) | Second MT task? | Experi-mental task? | Ref. |
| --- | --- | --- | --- | --- | --- | --- | --- | --- | --- | --- | --- | --- |
| OCD/traits | Einstein and Menzies (2004a) | 61 | 33, 17-62 | 56 | / | / | OCD | Mean, SD | TAFS 3 | Y | N | 3 |
|  | Farrell & Barrett (2006) | 34+39+38 | 9.6(1.6), 6-11; 14.1(1.6), 12-17; 32.2 (12.5), 18-66 | 44; 59; 63 | / | / | OCD, ANX | Mean, SD | TAFS 3 | Y | Y (ICA) | 208 |
|  | Nelson et al (2006) | 71 | 34.7(10.7) | 48 | / | MS, EDU, RELIG | OCD, ANX | Mean, SD ranges | TAFS 2 | Y | N | 43 |
|  | Zucker et al (2006) | 42+43 | 18.8(1.1), 18-22 | 73 | Y 29% Asian | / | OCD, DEP | Mean, SD | TAFS 2 | N | N | 56 |
|  | Samuels et al (2007) | 235+389 | 37, 9-89; 35.2 (7-95) | 68; 66 | / | MS | OCD, PD etc | YBOCS n tot count | SPQ MT SUB TOT | N | N | 66 |
|  | Verhaak and De Haan (2007) | 39 | 8-12; 13-18 | 56 | Partial - “Majority CAU” | SES | OCD | Mean, SD | Y for MTQ | CYBOCS (sub not rep) | N | 29 |
|  | Einstein and Menzies (2008) | 34 | 34, 17-51 | 65 | / | / | OCD | Mean, SD | Y MIS minus OCD items | N | N | 57 |
|  | Storch et al (2009) | 87 | 34(11) | 48 | Y 96% CAU | / | OCD, ANX | Mean, SD | TAFS TOT | Y | N | 54 |
|  | Twohig et al (2009) | 4 (2 prs twins) | 33, 23 | 50 | Partial data | MS | OCD, DEP | Total score | TAFS TOT | Y | N | 213 |
|  | Calleo et al (2010) | 69 | 31(10.3) | 52 | Y 88% CAU | / | OCD, ANX, DEP | Mean, SD | TAFS TOT | N | N | 214 |
|  | Twohig et al (2010) | 79 | 37(15.5), 18-67 | 61 | Y 88.6% CAU | MS | OCD, MD, ANX | Mean, SD | TAFS 2 | N | N | 215 |
|  | Besiroglu et al (2011) | 55 | 27.7(8.5) | 58 | / | MS, EDU | OCD, DEP | Mean, SD | TAFS 2 | N | N | 216 |
|  | Einstein et al (2011) | 2 | 45; 25 | 50 | / | RELIG | OCD, DEP | Mean, SD | TAFS 3 | Y | N | 217 |
|  | Jonsson et al (2011) | 70 | 32.2(10) | 71 | / | MS, OCC | OCD, ANX, DEP | Mean, SD | TAFS 2 | N | N | 58 |
|  | Farrell et al (2012) | 46 | 11.3(2.9), 7-17 | 34 | / | / | OCD | Mean, SD | TAFS 3 | N | N | 32 |
|  | Fernandez de la Cruz et al (2013) | 383 | 14.3(2.2), 7-18 | 47 | / | / | OCD, DEP | YBOCS counts | CYBOCS SUB | N | N | 30 |
|  | Selles et al (2014) | 99; 193 | 7.5(1.4), 3-9; 12.8(2.1), 10-18 | 45; 52 | 89% CAU | / | OCD | Mean, SD | YBOCS SUB | N | N | 31 |
|  | Ekinci and Ekinci (2016) | 84 | 30.5(10.5) | 68 | / | OCC, SES | OCD, ANX, DEP | Mean, SD | TAFS 2 | N | N | 36 |
|  | Kim & Lee (2020) | 65; 45 | 22.9(3.5), 18-30; 22.6(2.0), 18-30 | 20; 9 | / | / | OCD, DEP | Mean, SD | TAFS 3 | N | N | 62 |
|  | Coughtrey et al (2021) | 177; 54 | 34.3(11.4), 18-65; 33.4(10.9) | 73; 65 | / | / | OCD, ANX, DEP | Mean, SD | TAFS 3 | T TAFS and TAFS-C | N | 41 |
|  | Benatti et al (2022) | 70 | 21.7(9.0) | 44 | / | MS, OCC, EDU | OCD | Rates no table | YBOCS SUB | N | N | 55 |
|  | Lee et al (2022) OCD FC | 41 + 47 | 25.3(6.5); 22.6(1.9), 18-35 | 12; 2 | / | / | OCD, DEP | Mean, SD | TAFS TOT | Y | Y IND (NRR) | 70 |
|  | Lee et al (2022) | 32 + 38 | 25.3(7.2); 23.0(2.1), 18-46 | 0 | / | / | OCD, DEP | Mean, SD | TAFS TOT | Y | Y IND (NRR) | 72 |
|  | Burhan et al (2023) | 56 | 32.1(9.9) | 23 | / | MS | OCD, ANX | Change no means | TAFS TOT | N | N | 59 |
|  | Hansmeier et al (2023) | 88 | 26.5(12.0) | 94 | 100% CAU | / | OCD | Mean, SD | TAFS TOT | Y | Y IND | 42 |
|  | Lee et al (2023) | 93 + 45 | 25.4(6.0). 24.0(3.5) | 30; 18 | / | / | OCD | Mean, SD | TAFS TOT | Y | Y IND (NRR) | 44 |
|  | Cetin et al (2024) | 37 + 36 | 32.4(11.9); 28.6(6.4) | 65; 56 | / | MS, OCC | OCD, ANX, DEP, SSD | Median and range | TAFS 3 | Y | N | 67 |
|  | Farouk et al (2024) | 40 | 24.6(5.0) | 45 | / | MS, EDU OCC | OCD, AQ, SSD | YBOCS/SPQ reg only | SPQ MT SUB TOT | N | N | 68 |
|  | Lee et al (2024) ACT | 21 + 21 | 24.9(5.1); 26.5(6.4) | 43; 29 | / | / | OCD, DEP | Mean, SD | TAFS TOT | Y | Y IND (NRR) | 73 |
| SSDs/traits | Sobin et al (2000) | 119 | 34.2(10.1) | 55 | / | / | OCD, tics, SSD | Only counts | SIS | N (YBOCS MT not reported) | N | 97 |
|  | Chang and Lenzenweger (2001) | 39; 30 | 26.6(6.5), 18-45 | 56; 53 | 65% CAU | EDU | SSD | Mean, SD | SPQ MT SUB TOT | N | N | 81 |
|  | Torgersen et al (2002) | 663 | 45(11.9), 32-69; 44.1(13.7), 21-77; 52.1(14.3), 19-87, 52.9*15.5), 19-87. | 36, 59, 63, 47 | / | / | SSD, BPD | Mean std scores | SPQ MT SUB TOT | N | N | 111 |
|  | Mason et al (2004) | 74 | 17.3(2.8) | 47 | / | IQ, EDU | ANX, DEP, SSD | Count | Single score | N | N | 102 |
|  | Berle et al (2006) | 27;27 | 33.6(8.3), 20-49; 23.7(2.6) | 30; 30 | / | EDU | OCD, SSD (delu) | Mean, SD | TAFS 3 | Y | N | 201 |
|  | Yung et al (2006) | 140 | 17.7 | 58 | / | / | ANX, DEP, SSD | Counts, Mean SD | CAPE MT factor | N | N | 110 |
|  | Kabakci et al (2008) | 45;41 | 36.6(12.5), 19-65 | 46 | / | EDU | OCD, DEP, SSD | Mean, SD | TAFS 3 | Y | N | 101 |
|  | Bedwell et al (2013) | 28 | 18.9(1.2), 18-22 | 39 | 82% CAU | / | SSD /PD screen | Corr/reg only | SPQ MT SUB TOT | N | N | 94 |
|  | Garcia-Montes et al (2014) | 37; 30; 37; 41; 42 | 35.4; 36.7; 31.3; 31.5; 35.1 | 42 | / | / | DSM screen | Mean, CI | MIS: each item | N | N | 8 |
|  | Bedwell et al (2018) | 135 | 37.8 (10.4) | 53 | 67% CAU | EDU IQ | SSD only | Mean, SD | SPQ MT SUB TOT | N | N | 96 |
|  | Saarinen et al (2022) | 1292 | 42.8(5.1) | 57 | / | EDU, SES, LE | MH screen | Mean, Reg | TCI | N | N | 113 |
|  | Speck and Witthoft (2022) | 410 | 27.5(8.5), 18-68 | 78 | / | MS, EDU | SSD, ANX | Mean, SD | SPQ MT SUB SUB | N | N | 80 |
| Anxiety and mood disorders | Rassin et al (2001) | 44 | 32.8(9.2), 18-58 | 73 | / | / | OCD | Mean, SD | TAFS 3 | N | N | 128 |
|  | Abramowitz et al (2003) | 20; 19; 17; 20;19;25 | 34.9(10.4); 37.8(12.0); 42.7(14.8); 36.3(12.5)37.8(13.4); 35.5(9.3) | 47; 46; 41; 39; 53; 48 | / | / | OCD, DEP, ANX | Mean, SD | TAFS 3 | N | N | 200 |
|  | Barrett & Healey (2003) | 28; 17; 14 | 10.7(1.9); 9.2(2.2; 9.9(1.7) | 50; 53; 43 | / | / | OCD, ANX | Mean, SD | Own Questions | N | N | 226 |
|  | Einstein and Menzies (2006) | 20;11;19;21 | 38.3(12.1); 34.7(10.1); 40.4(12.6); 37.7(13.7) | 65; 64; 68; 14 F | / | NAT | OCD | Mean, SD | MIS TOT | N | N | 228 |
|  | O'Leary et al (2009) | 20; 21 | 32.1(11.(); 32.2(10.9) | 60; 71 | / | / | OCD, DEP | Mean, SD | TAFS 3 | N | N | 229 |
|  | Hausteiner-Wiehle and Sokollu (2011) | 61; 140 | 42; 45 33-54 | 79; 69 | / | CH; EDU, OCC | SSD, DEP, ANX | Counts, median | SPQ each item | N | N | 129 |
|  | West & Willner (2011) | 40; 15; 19 | 36.5(12.5), 18-65; 38.8(13.2), 23-62; 42.8(9.4), 21-57 | 60; 67; 74 | / | / | OCD, ANX | Mean (fig) | MIS TOT | N | N | 126 |
|  | Brown & Naragon-Gainey (2013) | 700 | 32.4(11.6), 18-74 | 61 | 91% CAU | / | OCD, DEP, ANX | Corr/reg only | TAFS TOT | N | N | 123 |
|  | Ghamari-Kivi et al (2013) | 50; 50 | 26.4(5.8); 29.8(8.1) | 80; 84 | / | / | OCD, DEP | Mean, SD | TAFS 3 | N | N | 120 |
|  | Thompson-Hollands et al (2013) | 37 | 29.7(9.4), 19-52 | 58 | 97% CAU | / | SSD, ANX, DEP | Mean, SD | TAFS 2 | N | N | 125 |
|  | Gjelsvik et al (2018) | 130; 134;97 | 36.1(12.8); 35.6(9.4); 38.2(11.4) | 92; 57; 71 | 89% 63% 91% WH | OCC, EDU | MH screen | Mean, SD | TAFS L only | N | N | 122 |
|  | Arnaez et al (2021) | 479; 31 | 30.6(14.2), 18-65; 32.7(9.7), 18-65 | 66; 52 | / | MS, EDU, SES | MH screen | Mean, SD | IIT SUB | N | N | 127 |
|  | Olivares-Olivares et al (2022) | 130 | 29.6(11.8), 17-62 | 71 | / | MS, EDU | ANX, DEP | Mean, SD | OBIR (S) SUB | N (YBOCS MT not reported) | N | 230 |
|  | Lee et al (2024) | 37; 37 | 25.4(5.3)26.8(5.3) | 59; 51 | / | EDU | OCD, ANX,DEP | Mean, SD | TAFS 2 | Y | Y IND (NRR) | 121 |
|  | Shams et al (2024) | 50; 50; 130 | 30.5(6.9); 36.5(11.3); 30.5(9.3) | 64; 64; 52 | / | EDU | MH screen | Mean, SD | IITIS tot | N | N | 124 |
|  | Lee et al (2025) | 34; 43; 46 | 25.4(5.1);26.4(6.4);27.9(6.1) | 68; 49; 52 | / | EDU | OCD, DEP | Mean, SD | TAFS 2 | N | N | 119 |
| Eating Disorders | Radomsky et al (2002) | 30;27;24;410 | 36.1(11.0); 43.8(14.9); 38.1(14.5); 22.5(4.5) | 57; 44; 63; 86 | / | / | OCD, ANX, DEP | Mean, SD | TAFS TOT | Y | N | 130 |
|  | Roncero et al (2011) | 15; 21; 18; 18; 22 | 24.8(7.3), 16-45; 21.8(5.3), 15-39; 25.8(9.1), 14-47; 22.7(5.4), 15-33; 33.8(11.4), 15-60. | 100 | / | MS, SES | OCD | Mean, SD | TAFS 2 | N | N | 131 |
|  | Garcia-Soriano et al (2014) | 79;177 | 34.8(12.0); 26.7(9.3) | 52; 100 | / | SES | OCD | Mean, SD | TAFS 2 | N | N | 133 |
|  | Lee et al (2020) | 112 | 18.9(5.7), 12-45 | 100 | / | / | OCD | Mean, SD | TAFS 3 | N | N | 132 |
| PDs: Borderline personality disorder | Zanarini et al (2013) | 290 | 27(6.3) | 77 | 87% WH | SES | MH screen | Counts | BPD IV | N | N | 138 |
|  | Alesiani et al (2014) | 32 | 44.4(9.3)26-63 | 81 | / | / | MH screen | Mean, SD | TCI-ST | N | N | 140 |
| Early trauma | Selvi et al (2012) | 95 | 27.8(8.5),18-55 | 66 | / | MS, EDU | OCD, DEP | Mean, SD | TAFS TOT | N | N | 177 |
|  | Velikonja et al (2019) | 225; 127 | 36.6(11.7), 18-64; | 46 | 41% WH | EDU, NA | MH screen, SSD | Means (in fig) | SPQ MT SUB TOT | N | N | 176 |

**KEY**

ABBREVIATIONS: /= Data not available; ANX: Anxiety Disorder; BPD IV: BPD interview; CAPE: Community Assessment of Psychic Experiences; CATH: Catholic; CAU: Caucasian; CH/LE: Co-habitation/Living environment; CORR/REG: Correlation/regression data; C-YBOCS: Childrens Yale-Brown Obsessive Compulsive Scale; DEP: Depressive Disorder; EDU: Education level; ICA: Idiopathic Cognitive Assessment; IITIS: International Intrusive Thoughts Schedule; IITI; Ilness Intrusive Thoughts Inventory; IND: TAF induction type task; IQ: Intelligence Quotient; OCD: Obsessive Compulsive Disorder; PD: Personality Disorder; BPD: Borderline Personality Disorder; MH: Mental Health; MIS: Magical Ideation Scale; MS: Marital Status; MT: Magical Thinking; MTQ: Magical Thinking Questionnaire; N: No, criterion not fulfilled; NAT: Nationality; NRR: No results reported; OBIR(S): Obsessive Beliefs Inventory Revised in Spanish; OCC: occupation; RELIG: religion; SD: Standard Deviation; SE: Standard Error; SES: Socio-Economic Status; SIS: Structured Interview for Schizotypy; SPQ: Schizotypal Personality Scale; SSD: schizophrenia/schizotypy; SUB: Subscale/s; TAFS: Thought Action Fusion Scale; TAFS-L: TAFS Likelihood Subscale; TAFS-LO: TAFS Likelihood Subscale for Other; TAFS-LS: TAFS Likelihood Subscale for Self; TAFS–M: TAFS Moral Subscale; TAFS-2: refers to use of the TAF-M and TAF-L subscales; TAFS-3: refers to use of TAFS-M, TAFS-LO and TAFS-LS subscales; TCI-ST; Temperament and Character Inventory Transcendental Subscale; TOT: Total (score); WH: White; Y: Yes, criterion fulfilled; YBOCS: Yale-Brown Obsessive Compulsive Scale. *Note: See Supplementary Table 1 key for additional references.*
