# Supplementary material for: The many manifestations of magical thinking: a systematic review
Source: Front Psychiatry. 2026 May 20;17:1759906. doi: 10.3389/fpsyt.2026.1759906 (PMC13230226; doi:10.3389/fpsyt.2026.1759906)
Supplement: Supplementary file 3 [file Table3.docx]

**Supplementary Table 3. Quality appraisal of nonclinical studies on the topic of MT**

| Topic | Study | N sample; subsamples | Age: Mean (SD), Range | % Female | Ethnicity data | Other sample data | MH scales or screen | MT data presented | MT scores detail (Inc. TAFS specific) | Second MT task? | Experi-mental task? | Ref. |
| --- | --- | --- | --- | --- | --- | --- | --- | --- | --- | --- | --- | --- |
| OCD/traits | Rachman et al (1996) | 63 | 19.5, / | 64 | / | / | DEP, OCD | Mean, SD | TAFS 3 | Y | Y | 206 |
|  | Shafran et al (1996) | 147 + 190; 122 + 272 | 38, / | 74 | / | / | OCD, DEP | Mean, SD | TAFS 3 | N | N | 2 |
|  | Emmelkamp & Aardema (1999) | 305 | 45(18), 19-86 | 65 | / | / | OCD, DEP | Mean, SD | TAFS TOT | N | N | 51 |
|  | Rassin et al (2000) | 20+24 | 32.8(9.2), 18-58 | 73 | / | / | OCD | Mean, SD | TAFS 3 | N | N | 48 |
|  | Smari & Holmstein (2001) | 211 | 25.6(7.6) | 65 | / | / | OCD | Mean, SD | TAFS TOT | N | N | 49 |
|  | Einstein and Menzies (2004b) | 86 | 22 | 62 | / | / | OCD | Mean, SD | TAFS 3 | Y | N | 207 |
|  | Gwilliam et al (2004) | 200 | 33.5(11.4), 18-69 | 68 | / | / | OCD, ANX | Corr/reg only | TAFS 3 | Y | N | 6 |
|  | Altin & Gencoz (2007) | 283 | 20.1(1.4), 17-24 | 57 | Y Turkish | / | OCD, DEP | Mean, SD | TAFS TOT | N | N | 46 |
|  | Belloch et al (2007) | 573; 75+25+22 | 27.4(11.1); 33.9(10.9; 32(9.2); 44.9(10.7) | 70; 53; 60; 82 | / | SES | OCD, DEP | Mean, SD | TAFS 2 | N | N | 50 |
|  | Bocci & Gordon (2007) | 51 | 20.7(4.3), 18-38 | 90 | / | / | OCD, ANX | Mean, SD | TAFS 2 | Y | Y IND | 209 |
|  | Marcks & Woods (2007) | 117 | 21.4(6.6),18-63 | 65 | Y 78% CAU | EDU | OCD | Mean, SD | TAFS 2 | Y | Y IND | 210 |
|  | Matthews et al (2007) | 223 | 13-16 | 66 | / | / | OCD | Mean, SD Plus range | TAFS 2 | N | N | 211 |
|  | Storch et al (2007) | 80 | 12.84/13.26 (2.8/3.3) | 1.27/.75 M:F 38% | / | / | OCD, tics etc | Counts | YBOCS (MT not reported) | N | N | 65 |
|  | Hanstede et al (2008) | 17 | 25.7, 19-41 | 71 | / | / | OCD | Mean, SD | TAFS TOT | N | N | 212 |
|  | O'Kearney & Nicholson (2008) | 192 | 22.5, 18-51 | 62 | / | / | OCD, DEP | Mean, SD | TAF L ONLY | N | N | 35 |
|  | Valentiner & Smith (2008) | 690 | 18.7(1.3) | 61 | Y 71% CAU | MS, SES | OCD | Corr/reg only | TAFS 2 | N | N | 25 |
|  | Rees et al (2010) | 137 | 27(12), M 22 | 65 | / | / | OCD | Mean, SD Plus range | TAFS 2 | Y | N | 4 |
|  | Yorulmaz et al (2010) | 98; 89 | 25(7.8), 18-50; 25.6(7.7), 18-48 | 56; 57 | / | NAT, RELIG | OCD | Mean, SD | TAFS 2 | N | N | 26 |
|  | Altin & Gencoz (2011) | 283 | 20.1(1.4), | 47 | / | NAT, EDU | OCD, DEP | Mean, SD | TAFS 2 | N | N | 47 |
|  | Coleman et al (2011) | 103 | 19.7(2.7) | 82 | 48% CAU | / | OCD, ANX | Mean, SD Plus range | TAFS 3 | N | N | 53 |
|  | Yorulmaz et al (2011) | 276 | 21.4(1.8) | 71 | / | NAT, RELIG | OCD, ANX, DEP | Mean, SD | TAFS 2 | N | N | 24 |
|  | Yorulmaz & Isik (2011) | 98; 89; 94 | 25.0(7.8), 18-50; 24.9(6.0), 18-42; 25.6(7.7), 18-48 | 56; 67; 54 | 65% Turkish | NAT, RELIG | OCD | Mean, SD | TAFS 2 | N | N | 27 |
|  | Aydin et al (2012) | 263 | 21.3(0.6), 17-40 | 46 | / | MS, urbanicity, SES | OCD | Corr/reg only | TAFS 2 | N | N | 218 |
|  | Helgadottir et al (2012) | 80 | 22.6(1.7) | 74 | / | NAT | OCD, DEP, ANX | Mean, SD | TAFS 3 | Y | N | 28 |
|  | McNicol & Wells (2012) | 177 | 22.1(5.3), 18-55 | 71 | / | / | OCD, ANX | Corr/reg only | TFI SUB | N | N | 219 |
|  | Berman et al (2013) | 47 | 20.6(2.2) | 64 | 68% CAU | / | / | Mean, SD | III/Ind SUB | Y | Y IND | 149 |
|  | Bailey et al (2014) | 548; 149 | 19.5(2.4); 29.2(14.3) | 52; 50 | 59% CAU; 89.3% CAU | / | OCD, ANX | Means, corr/reg | TAFS 3 | Y | N | 52 |
|  | Goods et al (2014) | 201 | 34.9(15.9)18-78 | 75 | / | NAT, OCC | OCD | Mean, SD Plus range | IBI TOT | N | N | 34 |
|  | Jones and Bhattacharya (2014) | 47 | 25.3(3.7) | 70 | / | / | OCD | Mean, SD | MIS TOT | Y | Y | 71 |
|  | Noorian et al (2015) | 966 | 13.9(0.1), 13-16 | 48 | / | NAT | OCD, DEP | Mean, SD | TAFQ SUB | N | N | 64 |
|  | Reuman et al (2017) | 290 | 19.95(1.7) | 63 | 74% WH | / | OCD, DEP, ANX | Mean, SD | TAFS 2 | N | N | 37 |
|  | Siwiec et al (2017) | 67 | 23(6.9) | 75 | 47% WH | MS | OCD, ANX, DEP | Mean, SD | TAFS 3 | N | N | 220 |
|  | Fergus & Rowatt (2018) | 320 | 19.1(1.2) | 72 | 58% WH | RELIG | OCD | Mean, SD | TAFS 2 | Y | Y | 40 |
|  | Jacoby et al (2018) | 304 | 18.6(1.3), 17-29 | 69 | 69.7% CAU | / | OCD | Mean, SD | CTAF TOT | N | N | 39 |
|  | Fite et al (2020) | 284 | 19.1(1.1) | 62 | 76.4% WH | / | OCD | Mean, SD | IBI TOT | N | N | 45 |
|  | Pennequin et al (2020) | 305(269) | 9.8(0.9), 8.2-11.7 | 51 | / | / | / | KIDCOPE 2 items mean | 2 items | N | N | 33 |
|  | Lee et al (2021) | 31 | 22.9(1.9) | 0 | / | / | OCD, DEP | Mean, SD | TAFS 3 | N | N | 69 |
|  | Marazziti et al (2021) | 25 | 42.3(13.5) | 28 | / | / | OCD, DEP | Just count | Only YBOCS | N | N | 221 |
|  | Cares et al (2022) | 70; 63; 101 | 19.5; 19.75; 19.5 | 63; 51; 41 | 51.4% WH; 40% WH; 50% WH | / | OCD, DEP, ANX | Mean, SD | TAFS 3 | Y | Y IND | 222 |
|  | Fite & Magee (2022) | 249 | 19.2(1.1) | 63 | 77% WH | / | OCD | Mean SD of 1 item | IBI subscales | Y | Y IND | 223 |
|  | Jellinek et al (2022) | 353 | 37.4(10.7) | 83 | / | / | / | Mean, SD | TAFS 3 | Y | Y IND | 224 |
|  | Ouellet-Courtois & Radomsky (2023) | 148 | 45.43(33.6) | 89 | 49% CAU | / | OCD, ANX, DEP | Corr/reg only | TAFS TOT | Y | Y IND | 38 |
| SSDs/traits | Raine (1992) | 787 (393+394) | / | 49 | / | / | SSD only | Mean (SPQ) | SPQ MT SUB TOT | N | N | 23 |
|  | Poreh et al (1993) | 96 | 19.3(13.5) | 0 | / | EDU | SSD only | SZ MAG means | MIS TOT | N | N | 92 |
|  | Roth and Baribeau (1997) | 257 | 21.5(1.9) | 53 | / | / | SSD only | Mean, SD | SPQ MT SUB TOT | N | N | 77 |
|  | Dickey et al (2003) | 20;11 | 37.1; 38.4 | 0 | / | SES, IQ | / | Corr/reg only | SCID MT score | N | N | 115 |
|  | Stefanis et al (2004) | 1411 | 20.9 (1.9) | 0 | / | IQ, urbanicity | SSD only | Mean, SD | SPQ MT sub TOT | N | N | 79 |
|  | Lee et al (2005) | 968 | 19.1(2.1), 16-47 | 68 | 62% CAU | / | SSD, ANX, DEP, OCD | Mean, SD | TAFS 3 | Y | N | 100 |
|  | Corlett et al (2009) | 50 | 21.1(1.1)18-22 | 64 | / | / | SSD / Delusions | Corr/reg only | MIS TOT | N | N | 89 |
|  | Yung et al (2009) | 881 | 15.6(0.5), 13.7-17.6 | 53 | / | / | SSD only | Mean, SD | CAPE MT factor | N | N | 109 |
|  | Steel et al (2009) | 384 | 24.9(7.2), 18-67 | 67 | 282 WH | / | DEP, ANX | Mean, SD | STA MT factor | N | N | 85 |
|  | Muris and Merckelbach (2010) | 77; 64 | 21.(1.9), 18-27; 19.5(1.7), 18-24 | 56; 86 | / | / | SSD only | Mean, SD | TAFS TOT | Y | N | 90 |
|  | Fonseca-Pedrero et al (2011) | 1438 | 15.9(1.2), 14-18 | 52 | / | / | SSD only | Mean, SD, counts | ESQUIZO-Q SUB | N | N | 99 |
|  | Armando et al (2012) | 997 | 21(2.45) | 76 | / | / | DEP, ANX | Mean, SD, counts | CAPE MT factor | N | N | 98 |
|  | Fonseca-Pedrero et al (2012) | 1618 | 15.9(1.2), 14-18 | 52 | / | / | SSD only | Mean, SD Plus range | ESQUIZO-Q SUB | N | N | 74 |
|  | Ribolsi et al (2013) | 205; 80 | 34.8(12.7), 32.2(11.1) | 66; 58 | / | / | MH screen | Mean, SD (supp tab) | SPQ MT SUB TOT | N | N | 95 |
|  | Collip et al (2013) | 512 | 15.6(2.6) | 51 | / | SES | DEP | Corr/reg only | CAPE MT factor | N | N | 84 |
|  | Barron et al (2014) | 447 | 23.2(7.9), 18-68 | 77 | / | NAT, EDU | SSD only | Mean, SD | SPQ MT SUB TOT | N | N | 103 |
|  | Dasse et al (2015) | 80 | 18-22 | / | / | / | / | Mean, SE | MIS TOT | N | N | 88 |
|  | Wiltink et al (2015) | 384 | 39.9(10.0) | 55 | / | / | DEP, PD | Counts CAPE | SCID, 1 item | N | N | 225 |
|  | Mededovic and Dordevic (2017) | 132; 119 | 19.2(1.8); 20.1(2.5) | 62; 69F | / | / | SSD, DEP | Mean, SD Plus range | DELTA-10 SUB | N | N | 91 |
|  | Barron et al (2018) | 411 | 35.4(14.1) | 61 | / | NAT | SSD only | Mean, SD | SPQ MT SUB TOT | N | N | 105 |
|  | Mimarakis (2018) | 445 | 18.0(1.1), 17-22 | 57 | 287 Greek | EDU, SES, setting | SSD, ANX | Mean, SD | STQ MT SUB | N | N | 78 |
|  | Weintraub et al (2018) | 170; 291 | 19.0(1.1),18-24; 35.5(11.0), 18-69 | 60; 56 | 72% CAU; 83% CAU | / | SSD, DEP, ANX | Corr/reg only | SPQ MT SUB TOT | N | N | 106 |
|  | March and Springer (2019) | 230 | 26.5(7.5) | 55 | / | NAT | SSD, NARC, PSYCHO | Mean, SD | SPQ MT SUB TOT | N | N | 104 |
|  | Turley et al (2019) | 587 | Year 10 | / | / | / | SSD, DEP | Corr/reg only | CAPE MT factor | N | N | 108 |
|  | Escola-Gascon et al (2020) | 174 | 28.8(7.9) | 57 | / | NAT/CITY | CAPE SSD DEP | Mean, SD | CAPE pseudosci beliefs SUB | N | N | 87 |
|  | Khaled et al (2020) | 1353 | Gives counts within ranges | 50 | 985 Arab | NAT, SES, MS, EDU | Distress | Counts | SPQ items | N | N | 75 |
|  | Dalal et al (2021) | 51 | 19.1(4.1), 17-41 | 47 | / | / | MH screen | Corr/reg only | SPQ MT SUB TOT | N | N | 82 |
|  | Eddy & Hansen (2021) | 297 | 19.2(1.2),18-29 | 85 | / | / | MH screen | Mean, SD | TAFS 3 | N | N | 83 |
|  | Elek et al (2021) | 346 | 21.5(1.8), 18-31 | 71 | 100% CAU | / | SSD only | Mean, SD, dist | SPQ MT SUB TOT | N | N | 112 |
|  | McDonald et al (2021) | 342 | 25.9(8.4), 18-61 | 75 | / | EDU | MH screen | Mean, SD (supp) | SPQ MT SUB tot | N | N | 93 |
|  | Chau et al (2022) | 2089 | 23.6(3.67), 18-30 | 68 | / | OCC, SES | ANX, DEP, SSD | Mean, SD Plus range | SPQ MT SUB TOT | N | N | 86 |
|  | Wastler and Lenzenweger (2023) | 205 | / | 81 | / | / | DEP | Mean, SD | MIS TOT | N | N | 107 |
|  | Garner et al (2024) | 257 | 42.5(14.9) | 81 | 88% WH | / | MH screen | Mean, SD | SPQ MT SUB TOT | N | N | 114 |
| Anxiety and mood disorders | Coles et al (2001) | 173 | STU / | 68 | 69 EA 49 AA | MS, RELIG | OCD, ANX | Mean, SD | TAFS 3 | N | N | 116 |
|  | Presson & Benassi (2003) | 85 | 18-19 | 100 | / | / | DEP | N | MIS TOT | N | N | 117 |
|  | Libby et al (2004) | 28; 28; 62 | 14.1(1.1) | 57; 79; 50 | / | / | OCD, ANX | Mean, SD | TAF L ONLY | N | N | 227 |
|  | Yorulmaz et al (2008) | 51; 44; 50 | 29.6(8.3);31.7(8.9);31.0(9.5) | 71; 71; 60 | / | MS, EDU, OCC | OCD | Mean, SD | TAFS 2 | N | N | 63 |
|  | Belloch et al (2010) | 75; 25; 22 | 33.9(10.9); 32.0(9.2); 44.9(10.7) | 53; 60; 82 | / | / | OCD, ANX, DEP | Mean, SD | OBIR(S) SUB | N | N | 118 |
| Eating Disorders | Pullmer et al (2020) | 86 | 15.6(1.61), 13-18 | 73 | / | / | OCD, DEP | Mean, SD | TAFS TOT | Y | N | 134 |
| Gambling compulsions | Teed (2012) | 118 | 19-25 | 67 | / | / | / | Mean, SD | GBQ SUB | N | N | 137 |
|  | Savage (2014) | 4764 | 37.7, 32-43 | 57 | / | MS, EDU, OCC, SES | / | N - LCA | MIS TOT | N | N | 136 |
|  | Passanisi (2017) | 222 | 19.7(3.1)19-21 | 47 | 100% CAU | / | / | Mean, SD | MIS TOT | N | N | 135 |
| Spirituality or religion | Rassin and Koster (2003) | 100 | 21.7(2.9), 18-43 | 85 | / | RELIG (CATH 60%) | OCD | Mean, SD | TAFS 3 | N | N | 142 |
|  | Yorulmaz et al (2009) | 115; 104 | 20.5(1.7); 20.0(1.9) | 73; 77 | / | NAT, RELIG, MS/CH | OCD | Mean, SD | TAFS 2 | Y | N | 147 |
|  | Berman et al (2010) | 43; 30 | 20.2(4.5), 18-47; 20.0(1.8), 18-25 | 79; 60 | 58; 67% CAU | RELIG (PRO, AG/ATH) | DEP | Mean, SD | TAFS 3 | Y | Y iND | 149 |
|  | Siev et al (2010) | 341 | 19.28 | 66 | 80% CAU | RELIG (MUS, JEW) | OCD | Mean, SD | TAFS 2 | N | N | 145 |
|  | Unterrainer et al (2011) | 102 | 23.8(3.0,)18-32 | 52 | / | / | / | Corr/reg only | MIS TOT | N | N | 152 |
|  | Berman et al (2013) | 407 | 19.4(1.7) | 68 | 71% WH | RELIG (43% PRO) | / | Mean, SD Plus range | TAFS 2 | N | N | 149 |
|  | Cougle et al (2013) | 139; 176 | 19.2(1.7),18-30; 19.3(3.1), 18-56 | 71; 77 | 64% CAU; 72% CAU | RELIG(mixed, 26% CATH, 27% ‘other’) | OCD, DEP | Mean, SD | TAFS 2 | Y | Y IND | 150 |
|  | Deacon et al (2013) | 60 | 35.8(11.7), 29-84 | 9 | 96% CAU | OCC, RELIG(PRO) | / | Mean, SD | TAFS 2 | N | N | 144 |
|  | Williams et al (2013) | 85 | 20.1(3.8), | 59 | / | RELIG (30% ATH/AG, 26% PRO) | OCD, DEP, ANX | Mean, SD | TAFS 3 | N | N | 151 |
|  | Fergus et al (2014) | 102; 128 | 35.7(11.6), 18-66; 38.8(13.2), 18-71 | 62; 61 | 78%; 85% CAU | RELIG (CATH; PRO), MS, OCC | OCD, SSD | Mean, SD | TAFS TOT | Y | N | 143 |
|  | Inozu et al (2014) | 244 | 20.6(2.0) | 75 | / | RELIG, NAT(MUS 95%) | OCD, DEP | Mean, SD | TAFS TOT | N | N | 159 |
|  | Breslin & Lewis (2015) | 371 | 28.9(11.0) | 78 | / | NAT | SZ, Bord | Mean, SD | STA SUB | N | N | 156 |
|  | Eremsoy & Inozu (2016) | 165 | 38.5(11.3), 23-67 | 50 | 87% Turkish | NAT, EDU, MS, SES, RELIG (91% MUS) | OCD | Corr/reg only | MIS TOT | N | N | 162 |
|  | Mauzay et al (2016) | 801 | 20.0(2.7), 18-51 | 73 | / | RELIG (45% CHR) | OCD, ANX, | Corr/reg only | TAFS 2 | N | N | 160 |
|  | Breslin & Lewis (2017) | 371 | 28.9(11.0)16-62 | 78 | / | / | SSD, BPD | Mean, SD | STA SUB | N | N | 157 |
|  | Siev et al (2017) | 34; 43 | 31.2(10.4); 28.9(5.1) | 50; 54 | / | EDU, RELIG(MUS; JEW) | OCD, DEP, ANX | Mean, SD | TAFS M ONLY | N | N | 146 |
|  | Jones et al (2019) | 64 | 38.8 (8.6), 25-69 | 0 | / | RELIG(MUS), OCC | / | Mean, SD | TAFS 2 | N | N | 231 |
|  | Kiken (2019) | 645;566 | 37.8(12.8); 37.5(11.9) | 56; 58 | 77%; 78% WH | EDU | / | Only Reg, range | IBI: MCB and TAF | N | N | 158 |
|  | Younce and Wu (2020) | 99 | 19.2(1.6) | 62 | 50% AA | RELIG (PRO) | OCD Screen | Corr/reg only | TAFS 2 | Y | Y IND | 161 |
|  | Fite et al (2021) | 594 | 39.7(12.1), 18-74 | 60 | 81% WH | RELIG (55% CHR), POL | / | Mean, SD | TAF M only | N | N | 155 |
|  | Henderson et al (2022) | 746; 24 | 30.5(13.9),17-65; 34.3(12.6), 18-61 | 68; 58 | 53%; 92% WH | RELIG (12% CATH; 42% CATH) | OCD, ANX | Mean, SD | TAFS 3 | N (YBOCS MT not reported) | N | 154 |
|  | Kumar et al (2025) | 734 | 20.3(3.5), | 60 | Indian | RELIG (80% HIN), SES | / | Mean, SD | SPQ MT SUB TOT | N | N | 153 |
|  | Namdiero-Walsh et al (2022) | 93; 98; 160; 135 | 25(6), 19-52; 21(2), 17-31; 22(3), 19-43; 22(2), 19-28 | 20; 24; 38; 18 | / | RELIG (CHR; HIN; MUS; BUD), NAT | / | Counts, prev | Ritual questions | N | N | 148 |
| Cognitive factors | Keinan (1994) | 174 | 37.4(14.9),18-83 | 58 | / | MS, EDU, OCC, NAT | / | Mean, SD | MTQ SUB | N | N | 165 |
|  | Zucker et al (2002) | 72 | 19.0(1.1), 18-23 | 68 | / | / | ANX | Mean, SD | TAFS 3 | Y | TAF IND | 170 |
|  | Beitel et al (2004) | 200 | 26.0(9.7) | 78 | 53% WH | / | / | Mean, SD | MIS TOT | N | N | 164 |
|  | Marino et al (2008) | 714 | 18.5(1.8) | 62 | 70% WH | RELIG | OCD | Mean, SD | TAFS 2 | Y | N | 163 |
|  | Marino Carper et al (2010) | 139 | 18-40 (% ranges provided) | 82 | 53% EA | / | / | Mean SE (in fig) | TAFS 2 | Y | Y IND | 169 |
|  | Ching and Tang (2016) | 140 | 20.7(1.6) | 64 | 110 SC | / | / | Mean, SD (in figs) | TAFS 3 | Y | Y IND | 171 |
|  | Zhu et al (2017) | 622 | 21.0(2.3)18-30 | 76 | 100% CHI | / | OCD, ANX, DEP | Mean, SD | TAFS 2 | N | N | 168 |
|  | Jimenez-Ros et al (2020) | 85 | 25.9(9.1), 18-51 | 53 | / | EDU | OCD, ANX, DEP | Mean, SD | OBI TAF SUB | N | N | 172 |
|  | Siev et al (2022) | 66 | 19.0(0.8) | 58 | 59% WH | RELIG | OCD, ANX, DEP | Mean, SD | TAFS 2 | Y | N | 167 |
|  | Rezaei et al (2023) | 612 | 29.9(6.1) | 87 | / | MS, EDU, FAM ENV | OCD | Mean, SD | TAFS TOT | N | N | 166 |
|  | Berman et al (2013) | 407; 107 | 19.4(1.7), 17-27; 21.4(1.5),18-25 | 68; 82 | 71% CAU; 79% CAU | RELIG | / | Mean, SD Plus range | TAFS 2 | Y | Y IND | 173 |
|  | Lu et al (2020) | 4360 | 12.91(1.) SE), 11-16 | 47 | / | NAT, FAM ENV | / | Mean, SD | CAPE SUB | N | N | 175 |
|  | Lee et al (2024) | 40 | 28.6(6.4), 18-40 | 48 | / | / | ANX, DEP | Mean, SD | TAFS 2 | Y | Y IND | 174 |
| Neurophysiological studies | Bell et al (2007) | 12 | 24.1(4.1), | 50 | / | / | / | Mean SD Plus range | MIS TOT | N | N | 19 |
|  | Brugger et al (2007) | 40 | 56 , 37-74 | 50 | / | EDU | / | Median | MIS TOT | N | N | 179 |
|  | Lee et al (2019) | 32 | 23.5(1.5) | 0 | / | / | OCD, DEP | Mean, SD | TAFS 3 | Y | Y IND | 178 |
|  | Narmashiri et al (2022) | 20 | 22.5(4.1) | 50 | / | EDU | / | Mean, SD | PBSr TOT | N | N | 180 |
| Developmental | Bolton et al (2002) | 127 | 5-17, groups given | 48 | / | / | ANX | Mean, SD Plus median | MTQ 2/3 SUB | N | N | 181 |
|  | Evans et al (2011) | 313 | 10.2(1.9), 7-14 | 44 | / | / | ANX | Mean SD Plus range median | TAFIC SUB | N | N | 182 |

**KEY**

/: Data not available; AA: African American; ANX: Anxiety Disorder; ATH/AG: Atheist/agnostic; BPD IV: BPD interview; BUD: Buddhist; CAPE: Community Assessment of Psychic Experiences; CATH: Catholic; CAU: Caucasian; CH/LE: Co-habitation/Living environment; CHI: Chinese; CORR/REG: correlation/regression data; CHR: Christian; C-YBOCS: Childrens Yale-Brown Obsessive Compulsive Scale; DEP: Depressive Disorder; EDU: Education level; EA: European American; ESQUIZO-Q: Oviedo Questionnaire for Schizotypy Assessment; GBQ: Gambling Behaviour Questionnaire; HIN: Hindu; IBI: Illusory Beliefs Inventory; ICA: Idiopathic Cognitive Assessment; IITIS: International Intrusive Thoughts Schedule; IITI; Ilness Intrusive Thoughts Inventory; IND: TAF induction type task; IQ: Intelligence Quotient; OCD: Obsessive Compulsive Disorder; PD: Personality Disorder; BPD: Borderline Personality Disorder; MH: Mental Health; MIS: Magical Ideation Scale; MS: Marital Status; MT: Magical Thinking; MTQ: Magical Thinking Questionnaire; MUS: Muslim; N: No, criterion not fulfilled; NAT: Nationality; NRR: No Results Reported; OBIR(S): Obsessive Beliefs Inventory Revised in Spanish; OCC: Occupation; PBSr: Paranormal Belief Scale revised; PRO: Protestant; RELIG: Religion; SC: Singaporean Chinese; SD: Standard Deviation; SE: Standard Error; SES: Socio-Economic Status; SIS: Structured Interview for Schizotypy; SPQ: Schizotypal Personality Scale; SSD: Schizophrenia Spectrum Disorders/traits (scale for); STA: Schizotypy personality scale; SUB: Subscale/s; TAFIC: Thought Action Fusion Inventory for Children; TAFS: Thought Action Fusion Scale; TAFS-L: TAFS Likelihood Subscale; TAFS-LO: TAFS likelihood subscale for other; TAFS-LS: TAFS likelihood subscale for self; TAFS–M: TAFS Moral Subscale; TAFS-2: refers to use of the TAF-M and TAF-L subscales; TAFS-3: refers to use of TAFS-M, TAFS-LO and TAFS-LS subscales; TCI-ST; Temperament and Character Inventory Transcendental Subscale; TOT: Total (score); WH: white; Y: Yes, criterion fulfilled; YBOCS: Yale-Brown Obsessive Compulsive Scale. *Note: See Supplementary Table for additional references.*
