# Supplementary material for: The many manifestations of magical thinking: a systematic review
Source: Front Psychiatry. 2026 May 20;17:1759906. doi: 10.3389/fpsyt.2026.1759906 (PMC13230226; doi:10.3389/fpsyt.2026.1759906)
Supplement: Supplementary file 4 [file SupplementaryFile1.docx]

**Identification of studies via databases and registers**

Records removed *before screening*:

Duplicate records removed (n = 2)

Records identified:

Web of Knowledge databases (n = 427)

**Identification**

Records screened

(n = 425)

Records excluded as off topic, no automation

(n = 99)

Reports sought for retrieval

(n = 326)

Reports not retrieved

(n = 0)

**Screening**

Reports excluded:

Reason 1: review/theory paper (n = 25)

Reason 2: psychometric testing only (n = 48)

Reason 3: no formal MT scale used or no such data presented within results (n = 61)

Reason 4: full article not available in English (n=1)

Reports assessed for eligibility

(n = 326)

Studies included in review

(n = 191 )

Reports of included studies

(n = 191 )

**Included**

Source: Page MJ, et al. BMJ 2021;372:n71. doi: 10.1136/bmj.n71.

This work is licensed under CC BY 4.0. To view a copy of this license, visit <https://creativecommons.org/licenses/by/4.0/>
